# Total synthesis of the endogenous inflammation resolving lipid resolvin D2 using a common lynchpin

**DOI:** 10.3762/bjoc.9.310

**Published:** 2013-12-03

**Authors:** John Li, May May Leong, Alastair Stewart, Mark A Rizzacasa

**Affiliations:** 1 School of Chemistry, The Bio21 Institute, The University of Melbourne, Parkville, Victoria 3010, Australia; 2 Department of Pharmacology and Therapeutics, The University of Melbourne, Parkville, Victoria, 3010, Australia

**Keywords:** catalysis, eicosanoid, natural product, resolvin D2, Sonogashira coupling, total synthesis, Wittig reaction

## Abstract

The total synthesis of the endogenous inflammation resolving eicosanoid resolvin D2 (**1**) is described. The key steps involved a Wittig reaction between aldehyde **5** and the ylide derived from phosphonium salt **6** to give enyne **17** and condensation of the same ylide with aldehyde **7** to afford enyne **11**. Desilylation of **11** followed by hydrozirconation and iodination gave the vinyl iodide **4** and Sonogashira coupling between this compound and enyne **3** provided alkyne **18**. Acetonide deprotection, partial reduction and ester hydrolysis then gave resolvin D2 (**1**).

## Introduction

**Figure 1:**
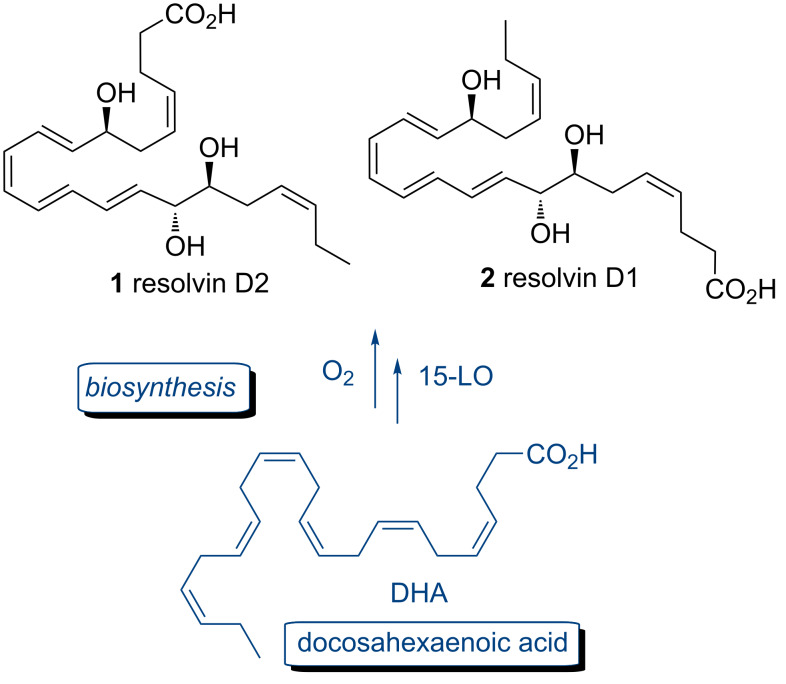
Structures of resolvins D1 (**1**) and D2 (**2**).

The resolution of inflammation is a tightly governed active process effectively mediated by a range of bioactive polyunsaturated fatty acids, peptides and proteins. In 2002, a new family of endogenously generated lipid mediators involved in the resolution of inflammation named the resolvins (resolution phase interaction products) were identified by Serhan and co-workers in the inflammatory exudates of aspirin treated mice [1–3]. The resolvins are divided into 2 groups, the D-series resolvins D2 (**1**) and D1 (**2**) [3], which are derivatives of docosahexaenoic acid (DHA) (Figure 1) and the E-series [4] derived from eicosapentaenoic acid. Structural analysis by mass spectrometry (MS) showed that resolvin D2 (RvD2, **1**) was a 17-hydroxy derivative of DHA (17HDHA) [1]. However, no NMR experiments were performed due to nanogram quantities isolated and the stereochemistry was tentatively assigned based on the proposed biosynthesis via lipoxygenase modification of DHA.

RvD2 (**1**) prevents the adherence of polymorphonuclear leukocytes (PMN) to the blood vessel wall by promoting the shedding of L-selectin from PMNs thus preventing binding to E selectin on the endothelial cell lining of the blood vessel [5]. Furthermore, RvD2 (**1**) promotes the influx and phagocytic activity of macrophages, facilitating clearance of dead cells and microbial pathogens, allowing resolution of inflammation and infection [5]. This successful evaluation of the resolvin series in preclinical models of bacterial sepsis has stimulated strong interest in their therapeutic potential, as RvD2 appears to express the unusual combination of anti-inflammatory and antimicrobial activity. Further interest in inflammation-resolving lipids is stimulated by their inhibitory effects on inflammatory pain, which are mediated via inhibition of the activity of TRPV1 and TRPA1 calcium channels on sensory nerves [6]. Resolvin D1 (**2**) [7] has been shown to act directly on human PMNs and also regulates actin polymerization [8]. Whilst RvD1 has been shown to act on the FPR2 and GPR32 types of G-Protein-coupled receptors, the receptor(s) for RvD2 remain to be identified. Identification of the receptors mediating the combined anti-inflammatory and antimicrobial actions would facilitate efforts to identify ligands that have better drug-like properties than RvD2 or its analogues. However, such efforts have been limited by the lack of availability of suitable amounts of RvD2.

The first total synthesis of (7*S*,16*R*,17*S*)-RvD2 (**1**) was communicated by Spur in 2004 [9] but this report did not include an experimental section although physical data for some compounds was provided. A similar synthesis of RvD2 was utilized by others for the production of **1** for a biological study [5] but again, there was no experimental provided. The total synthesis of resolvin D1 has also been reported [10] along with resolvins D3 [11], D5 [12], D6 [13] and resolvins E1 [4,14–15], E2 [16–17] and E3 [18] with full experimental details included for resolvins D3 [11], E2 [16] and E3 [18]. An improved synthesis of the C16–C20 fragment of resolvin E1 has also been reported [19]. We were interested in accessing amounts of RvD2 (**1**) for biological evaluation but without detailed synthetic sequence to follow and given the very high cost [20] of commercial **1** we elected to develop an alternative route to provide this important compound and analogues for further biological evaluation. Herein we describe a synthesis of RvD2 (**1**) which includes full experimental details so that other researchers can produce useful amounts of this important compound as well as novel isomers.

## Results and Discussion

### Retrosynthetic analysis

**Scheme 1:**
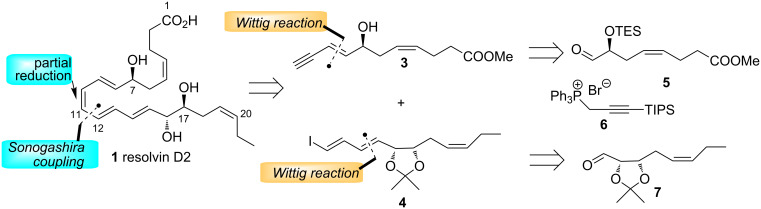
Retrosynthetic analysis of RvD2 (**1**).

A retrosynthetic analysis of RvD2 (**1**) is shown in Scheme 1. It was envisaged that the target compound **1** could be secured via a Sonogashira coupling to form the C11–C12 bond followed by partial reduction. This is similar to the endgame of the reported syntheses of **1** [5,9] but both of these approaches involved formation of the C9–C10 bond as the convergent step. In our approach, **1** could arise from enyne **3** and vinyl iodide **4** which could both be obtained by Wittig extension using the common linchpin phosphorus ylide derived from phosphonium salt **6** [21–22] and each of the homochiral aldehydes **5** and **7** [9].

### Synthesis of vinyl iodide **4**

**Scheme 2:**
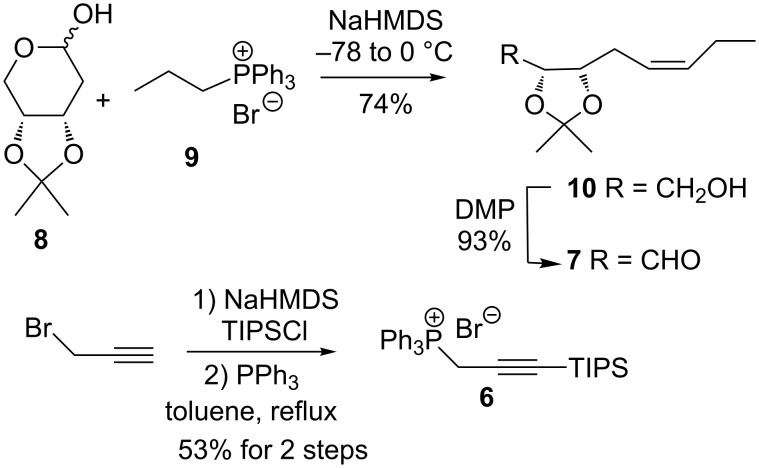
Synthesis of aldehyde **7** and phosphonium salt **6**.

The synthesis of fragment **4** began with the production of the aldehyde **7** as shown in Scheme 2. A Wittig reaction between hemiacetal **8** [23] and the ylide derived from **9** provided the alkene **10** [9] with excellent stereoselectivity. Oxidation of **10** with Dess–Martin periodinane then afforded aldehyde **7**. The phosphonium salt **6** [21–22] was produced from propargyl bromide via silylation of the derived sodium salt with TIPSCl followed by reaction with triphenylphosphine.

**Scheme 3:**
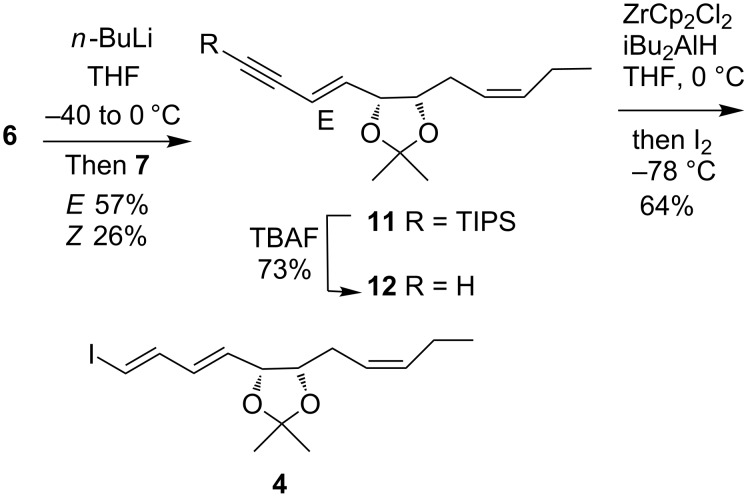
Synthesis of vinyl iodide **4**.

Treatment of the salt **6** with *n*-BuLi gave the ylide and condensation with the aldehyde **7** afforded the desired *E*-enyne **11** along with the *Z*-isomer in a ratio of 2.2:1 which were easily separated by flash chromatography (Scheme 3). The minor *Z*-isomer could also provide novel stereoisomer analogues of RvD2 (**1**). Removal of the TIPS group with TBAF gave terminal alkyne **12**. Alkyne **12** then underwent smooth hydrozirconation utilizing the procedure reported by Negishi [24] were ZrCp_2_HCl is generated in situ by reduction of ZrCp_2_Cl_2_ with DIBALH in THF. Iodinolysis of the zirconium species then gave the diene iodide **4** in good yield. Selectivity for this process was excellent with only a trace of the regioisomer formed.

### Synthesis of dienyne **3**

**Scheme 4:**
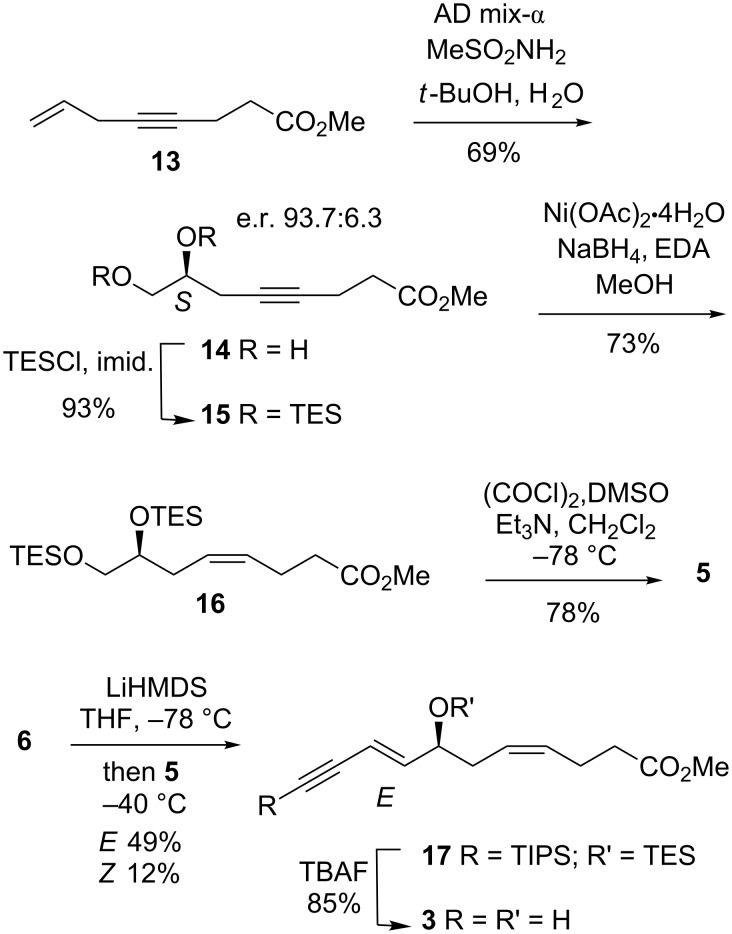
Synthesis of enyne **3**.

Our approach to the aldehyde **5** began with the production of the known bis-TES ether [9] produced by an alternative procedure (Scheme 4) in which the C7 stereochemistry was introduced via asymmetric dihydroxylation [25–26]. Thus, ester **13** [27] was treated with AD-mix-α in *t*-BuOH/H_2_O to give diol **14** in reasonable yield. The enantioselectivity and absolute configuration of the secondary alcohol was determined by conversion of diol into the bis-(*S*)-Mosher ester [28–29]. Integration of the ^1^H MMR spectrum indicated the e.r. was 93.7:6.3 and Mosher analysis (See Supporting Information File 1 for details) confirmed the stereochemistry of the new asymmetric center of the major enantiomer as *S* in accord with the predicted outcome [25]. Silylation gave bis-TES ether **15** and partial reduction of the alkyne using P2-Ni as catalyst [30] afforded the alkene **16**. Desilylation of the primary TES group in **16** and concomitant oxidation to aldehyde **5** was achieved under Swern conditions as reported by Spur [31].

Deprotonation of salt **6** with LiHMDS followed by condensation with aldehyde **5** gave the *E*-enyne and the corresponding *Z*-isomer in a 4:1 ratio. The use of LiHMDS as base was critical for reasonable yields and stereoselectivity in this case. Global deprotection of **17** with TBAF gave the enyne **3** in good yield.

### Total synthesis of resolvin D2 (**1**)

**Scheme 5:**
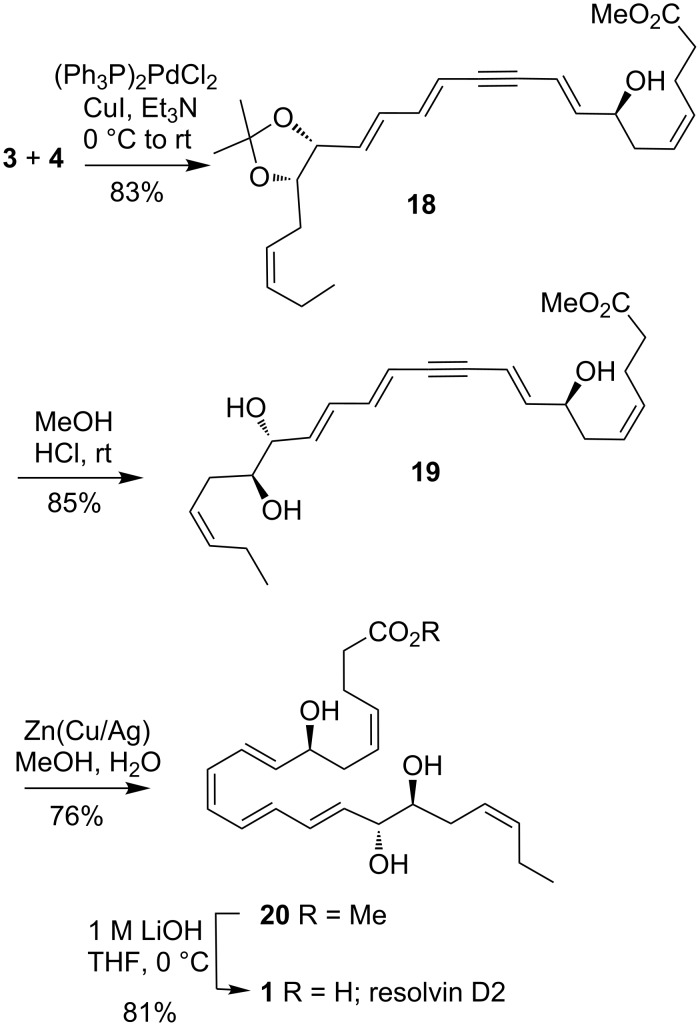
Completion of the synthesis of RvD2 (**1**).

The completion of the synthesis of RvD2 (**1**) is shown in Scheme 5. Sonogashira coupling [32–33] between **3** and **4** was very efficient giving the alkyne **18** in good yield. Removal of the acetonide was effected by treatment with HCl in MeOH to give the known triol **19** [9]. The final steps to **1** were similar with those previously reported [5,9]. Thus, partial reduction of the triple bond using Zn(Cu/Ag) [34] to afforded RvD2 methyl ester **20** in 76% yield. A large excess of the Zn reagent was required to obtain a good conversion of **19** into **20**. The ^1^H NMR spectrum (CDCl_3_ solvent) of RvD2 methyl ester (**20**) compared well to that reported [5]. Final ester hydrolysis and mild acid work-up then gave RvD2 (**1**).

The synthetic RvD2 (**1**) had physical data identical to that reported [9,35] and we measured the specific rotation of this material for the first time ([α]_D_ −17.5° (*c* 0.075, CH_2_Cl_2_)). In our hands, both RvD2 methyl ester (**20**) and RvD2 (**1**) itself were highly unstable, especially to acid. Prolonged standing in CDCl_3_ or CD_3_CN solution or exposure to light caused rapid decomposition and so NMR spectra were obtained quickly. We found that RvD2 methyl ester (**20**) was not very soluble in CD_3_CN so spectra were best run in CDCl_3_ that was filtered through basic alumina immediately prior to use. Spectra for RvD2 (**1**) were always measured for CD_3_CN. Even with short exposure to the solvent, we still observed degradation to unidentified compounds. Samples of RvD2 (**1**) can be stored in EtOH or frozen in DMSO solution but should be used immediately upon thawing. Alternatively, the triol **19** proved more stable than both RvD2 methyl ester (**20**) and RvD2 (**1**) and can be stored for longer periods prior to conversion to **1** which should be used rapidly for biological assessment to avoid degradation.

## Conclusion

The total synthesis of RvD2 (**1**) has been completed using a common linchpin Wittig reaction. Using this approach, we were able to prepare sufficient quantities of this important inflammation resolving compound for further biological evaluation.

## Supporting Information

File 1 Experimental.

File 2 ^1^H and ^13^C NMR spectra of all intermediates and the mass spectrum of RvD2 (**1**).
