# Supplementary material for: Total synthesis of the endogenous inflammation resolving lipid resolvin D2 using a common lynchpin
Source: Beilstein J Org Chem. 2013 Dec 3;9:2762–6. doi: 10.3762/bjoc.9.310 (PMC3869264; doi:10.3762/bjoc.9.310)
Supplement: File 1 — Experimental. [file Beilstein_J_Org_Chem-09-2762-s001.pdf]

Supporting Information File 1

for

Total synthesis of the endogenous inflammation resolving lipid resolvin D2 using a common lynchpin

John Li[1], May May Leong[1], Alastair Stewart[2] and Mark A. Rizzacasa*[1]

Address: [1]School of Chemistry, The Bio21 Institute, The University of Melbourne, Parkville, Victoria 3010, Australia and [2]Department of Pharmacology and Therapeutics, The University of Melbourne, Parkville, Victoria, 3010, Australia.

Email: Mark A. Rizzacasa* - masr@unimelb.edu.au

* Corresponding author

Experimental

General S2

Preparation of alcohol **10** S2

Preparation of phosphonium bromide **6** S3

Preparation of enyne **11** S4

Preparation of enyne **12** S5

Preparation of vinyl iodide **4** S6

Preparation of diol **14** S7

Preparation of bis-TES ether **15** S7

Preparation of alkene **16** S8

Preparation of enyne **17** S9

Preparation of enyne **3** S10

Preparation of alkyne **18** S11

Preparation of enyne triol **19** S12

Preparation of RvD2 methyl ester (**20**) S12

Preparation of RvD2 (**1**) S13

References S14

General

Proton nuclear magnetic resonance spectra (^{1}H NMR, 400 or 500 MHz) and proton decoupled carbon nuclear magnetic resonance spectra (^{13}C NMR, 125 MHz) were obtained in deuterochloroform or d_3-acetonitrile with residual protonated solvent as internal standard. Chemical shifts are followed by multiplicity, coupling constant(s) (*J*, Hz), integration and assignments where possible. Optical rotations were recorded for a 1 mL solution and units are deg·cm^{2}g^{-1}. Flash chromatography was carried out on silica gel 60. Analytical thin layer chromatography (tlc) was conducted on aluminium-backed 2 mm thick silica gel 60 GF_{254} and chromatograms were visualized with 20% w/w phosphomolybdic acid in ethanol. High resolution mass spectra (HRMS) were obtained by ionizing samples via electron spray ionization (ESI). Anhydrous THF, Et_2O and CH_2Cl_2 were used from the solvent cartridge system. Dry methanol was distilled from magnesium methoxide. All other solvents were purified by standard methods. Petrol used refers to petroleum ether 40–60 °C boiling range. All other commercially available reagents were used as received.

Alcohol 10

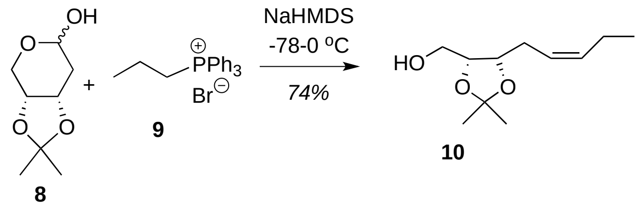

To a suspension of propyltriphenyl-phosphonium bromide **9** (12.2 g, 31.72 mmol) in THF (100 mL) at -78 °C was

added sodium hexamethyldisilylazide (1.0 M in THF, 7 mL, 7 mmol). The reaction mixture was stirred at −78 °C for 30 min before warming to 0 °C for 1 h with further stirring. The reaction was then cooled to −78 °C before the addition of the hemiacetal **8** (1.32 g, 7.6 mmol) in dry THF (45 mL). The mixture was slowly warmed up to room temperature with further stirring overnight. It was quenched by the addition of saturated aqueous NH_4Cl. The layers were separated and the aqueous layer extracted with Et_2O. The combined organic layers were washed with brine, water, dried ($MgSO_4$) and the organic solvent was removed under reduced pressure. Column chromatography of the residue using 20% EtOAc/petrol as the eluent afforded the alcohol **10** as a a yellow oil (1.10 g, 5.53 mmol, 74%). $[\alpha]_D$ +10.4 (*c* 1.0, CH_2Cl_2). ^{1}H NMR ($CDCl_3$, 400 MHz) δ 5.52 (dtt, *J* = 10.8, 6.8, 1.6 Hz, 1H, H19), 5.36 (dtt, J = 1.5, 7.0, 10.8 Hz, 1H), 4.19 (m, 2H), 3.65 (m, 2H), 2.38 (m, 1H) 2.28 (m, 1H), 2.06 (m, 2H), 1.88 (dd, J = 5.2, 6.8 Hz, 1H), 1.48 (s, 3H), 1.37 (s, 3H), 0.98 (t, J = 7.6 Hz 3H). ^{13}C NMR ($CDCl_3$, 125 MHz) δ 134.5, 123.9, 108.3, 77.9, 76.9, 61.8, 28.2, 27.4, 25.5, 20.9, 14.2.

Phosphonium bromide 6

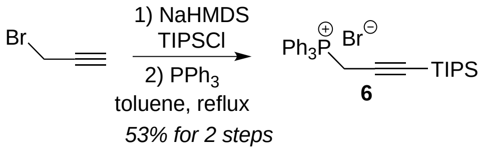

To a stirred solution of NaHMDS (6.0 mL, 1 M in THF, 2.0 mmol) in Et_2O (5 mL) at −78 °C was added a solution of propargyl bromide in toluene (80%, 0.54 mL, 5.0 mmol) and the mixture was stirred for further 5 min and TIPSCl (1.07 mL, 5 mmol) was added dropwise over 5 min. The mixture was stirred at −40 °C for 1 h before quenching with water and the aqueous layer extracted with Et_2O. The combined organic layers were washed with water, brine, dried ($MgSO_4$) and the organic solvent was removed under reduced pressure. Flash chromatography of the residue using petrol as the eluent afforded the product (1.04 g, 3.8 mmol, 76%) as colourless oil. A solution of triphenylphosphine (1.8 g, 7 mmol) and TIPS propargyl bromide (2.0 g, 7 mmol) in toluene

(4 mL) was heated under reflux overnight. The product was isolated by filtration and washed with petrol to afford salt **6** (2.7 g, 70%) as a light yellow powder. ^{1}H NMR ($CDCl_3$, 500 MHz) δ 7.93 (m, 2H), 7.80 (m, 1H), 7.67 (td, J = 7.79, 3.58 Hz), 5.27 (d, J = 15 Hz, 2H), 0.87 (m, 21H). HRMS (ESI) calc for $C_{30}H_{38}PSi$ [M]+: 457.24749 found 457.24738

(*E*)-Enyne 11

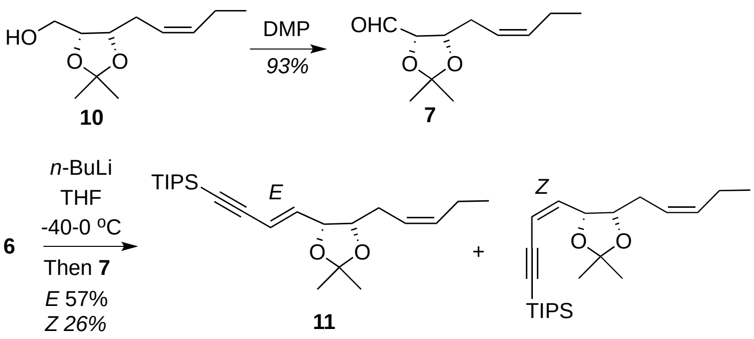

To a stirred solution of alcohol **10** (551.6 mg, 2.75 mmol) in CH_2Cl_2 (27mL) at 0 °C was added Dess–Martin periodinane (1.75 g, 4.12 mmol). After 1 h at 0 °C, the solution was quenched with 1:1 mixture of saturated $NaHCO_3$ and $Na_2S_2O_3$ (1.5 M) and the mixture was stirred until two clear layers formed. The layers were separated and the aqueous layer was extracted with CH_2Cl_2. The combined organic layers were washed with brine, water, dried ($MgSO_4$) and the solvent was removed under reduced pressure to afford the crude aldehyde **7** (507 mg, 2.55 mmol, 93%). To a suspension of the phosphonium salt **6** (2.1 g, 3.9 mmol) in THF (25 mL) at −78 °C was added *n*-BuLi (1.3 M, 3 mL, 3.6 mmol) dropwise. The resultant solution was warmed to −40 °C and a solution of the aldehyde (0.396 g, 2 mmol) in THF (15mL) was added via cannula. The reaction mixture was stirred further 30 min at −40 °C then at room temperature for 1 h and quenched by the addition of saturated aqueous NH_4Cl. The product was extracted with Et_2O and the combined organic layers were washed with brine, water, dried ($MgSO_4$) and the solvent was removed under reduced pressure. Column chromatography of the residue using 5% EtOAc/petrol as the eluent afforded the (*E*)-enyne **11** (430 mg, 57%). $[\alpha]_D$ +14.9 (*c* 1.16, CH_2Cl_2); ν_{max} (film) 2893, 2130, 1738, 1216, 1052, 882, 673 cm^{-1}; ^{1}H NMR ($CDCl_3$, 500 MHz) δ 6.11 (dd, J = 15.8, 7.3 Hz, 1H), 5.79 (dd, J = 15.8, 1.2 Hz, 1H), 5.49 (m, 1H),

5.31 (m, 1H), 4.54 (ddd, J = 7.3, 6.2, 1.1 Hz, 1H), 4.20 (m, 1H), 2.26 (m, 2H), 2.07 (m, 2H), 1.50 (s, 3H), 1.36 (s, 3H), 1.08 (m, 21H) 0.97 (t, J = 7.5 Hz, 3H). ^{13}C NMR ($CDCl_3$, 150 MHz) δ 139.41, 134.39, 123.74, 113.40, 108.67, 104.84, 92.29, 78.66, 78.48, 28.83, 28.18, 25.62, 21.05, 18.74, 14.24, 11.42. HRMS (ESI) calc for $C_{23}H_{40}O_2SiNa$ [M+Na]: 399.2690, found 399.2692.

Further elution gave the (*Z*)-enyne as a colorless oil (195 mg, 26%). $[\alpha]_D$ −17.7 (*c* 1.00, CH_2Cl_2); ν_{max} (film) 2866, 2147, 1216, 1055, 883, 677 cm^{-1}; ^{1}H NMR ($CDCl_3$, 500 MHz) δ 5.98 (dd, J = 11.0, 8.9 Hz, 1H), 5.72 (dd, J = 11.0, 1.1 Hz, 1H), 5.47 (m, 1H), 5.37 (m, 1H), 5.21 (ddd, J = 8.9, 6.3, 1.0 Hz, 1H), 4.24 (dt, J = 8.3, 5.9 Hz, 1H), 2.22 (m, 2H), 2.03 (m, 2H), 1.50 (s, 3H), 1.36 (s ,3H), 1.08 (m, 21H) 0.97 (t, J = 7.5 Hz, 3H). ^{13}C NMR ($CDCl_3$, 150 MHz) δ 140.21, 134.14, 124.31, 112.93, 108.67, 102.44, 97.71, 78.42, 76.65, 28.64, 28.30, 25.61, 20.91, 18.76, 18.74, 14.25, 11.38. HRMS (ESI) calc for $C_{23}H_{40}O_2SiNa$ [M+Na]: 399.2690, found 399.2691.

Enyne 12

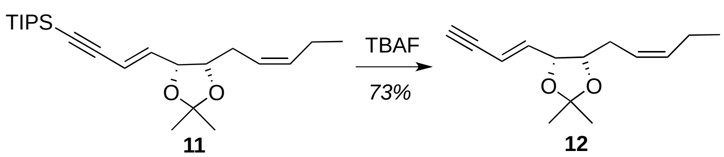

11 12

To a solution of enyne **11** (51 mg, 0.135 mmol) in THF (0.5 mL) at rt was added TBAF (35.3 mg, 0.14 mmol). The solution was stirred for 3 h then quenched with H_2O, extracted with CH_2Cl_2 washed with water, then brine and dried ($MgSO_4$). The crude product was then concentrated and purified via flash column chromatography using 10% EtOAc/Pet as the eluent to afford enyne **12** (21.8 mg, 73%) as a clear oil. $[\alpha]_D$ +11.3 (*c* 0.62, CH_2Cl_2); IR ν_{max} (film) 3295, 2935, 1370, 1216, 1055, 960 cm^{-1}; ^{1}H NMR ($CDCl_3$, 400 MHz) δ 6.20 (dd, J = 6.8, 15.6Hz, 1H), 5.76 (dd, J = 2.4, 16 Hz, 1H), 5.49 (m, 1H), 5.31 (m, 1H), 4.56 (t, J = 6 Hz, 1H), 4.19 (m, 1H), 2.92 (d, J = 2 Hz, 1H), 2.28 (m, 2H), 2.07 (m, 2H), 1.49 (s, 3H), 1.36 (s, 3H), 0.97 (t, J = 7.6 Hz, 3H). ^{13}C NMR ($CDCl_3$, 150 MHz) δ 141.0, 134.6, 123.7, 111.7,

108.8, 81.6, 78.5, 78.4, 78.4, 28.8, 28.2, 25.6, 21.0, 14.23; HRMS (ESI) calc for $C_{14}H_{20}O_2Na$ [M+Na]: 243.1356 found 243.1356.

Vinyl iodide 4

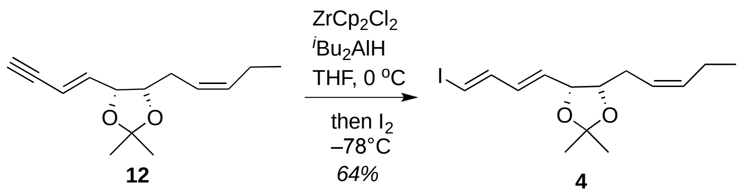

To a suspension of $ZrCp_2Cl_2$ (262.5 mg, 0.898 mmol) in THF (3.2 mL) at 0 °C was added a solution of iBu_2AlH in THF (1M, 0.89 mL, 0.898 mmol). The mixture was stirred for 30 min at 0 °C and a solution of enyne **12** (91.9 mg, 0.477 mmol) in THF (2.1mL) was added. The mixture was warmed to rt over 1 h and then cooled to −78 °C, followed by addition of I_2 (132.5 mg, 0.522 mmol) in THF (1 mL). After 1 h stirring at −78 °C, the solution was quenched by the addition of 1 M HCl. The layers were separated and the aqueous layer was extracted with Et_2O. The combined organic layers were washed with sat. $Na_2S_2O_3$, sat. $NaHCO_3$ and brine, dried ($MgSO_4$) and the solvent was removed under reduced pressure. Column chromatography of the residue using 5% EtOAc/petrol as the eluent afforded the vinyl iodide **4** as a brown oil (105.7 mg, 64%). $[\alpha]_D$ +5.61 (*c* 0.325, CH_2Cl_2); IR ν_{max} (film) 2960, 2873, 1380, 1369, 1216, 1164, 1105, 1016, 982 cm^{-1}; ^1H NMR ($CDCl_3$, 500 MHz) δ 7.05 (dd, J = 14.4, 10.7 Hz, 1H), 6.40 (d, J = 14.4 Hz, 1H, H12), 6.20 (dd, J = 15.2, 10.7 Hz, 1H), 5.70 (dd, J = 15.2, 7.6 Hz, 1H), 5.49 (m, 1H), 5.30 (m, 1H), 4.53 (dd, J = 7.1, 6.6 Hz, 1H), 4.18 (m, 1H), 2.21 (m, 2H), 2.02 (dt, J= 14.6, 7.2 Hz, 2H), 1.49 (s, 3H), 1.36 (s, 3H), 0.96 (t, J = 7.53 Hz, 3H). ^{13}C NMR ($CDCl_3$, 125 MHz) δ 144.4, 134.4, 132.8, 130.1, 124.0, 108.6, 80.5, 78.6, 78.4, 28.8, 28.3, 25.7, 21.0, 14.2; HRMS (ESI) calc for $C_{14}H_{21}O_2INa$ [M+Na]: 371.0478 found 371.0469.

Diol 14

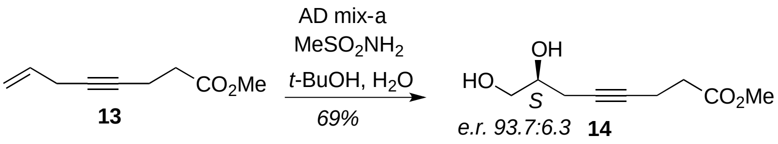

To a solution of AD-mix-α (3.86 g) in *t*-BuOH/H_2O (1:1, 20 mL) at 0 °C was added a solution of the methyl ester **13** [1] (4.2 g, 2.76 mmol) in *t*-BuOH/H_2O (1:1, 6 mL). After stirring at 0 °C for 2 h, Na_2SO_3 (4.14 g) was added and the mixture was warmed to room temperature over 1 h. The solution was extracted with EtOAc and the combined organic layers were washed with water, brine, dried ($MgSO_4$) and the organic solvent was removed under reduced pressure. The crude product was purified by flash chromatography with 70% EtOAc/petrol as eluent afforded the diol **14** [2] (3.54 g, 69%) as a white solid. [The e.e. was determined by complete conversion of **14** into the bis-(*S*) Mosher ester using 2.5 equiv DCC and 2.5 equiv (*S*)-(−)-α-methoxy-α-trifluoromethylphenylacetic acid followed by ^{1}H NMR analysis]. $[\alpha]_D$ +3.58 (*c* 0.13, CH_2Cl_2); ^{1}H NMR ($CDCl_3$, 500 MHz) δ 3.82 (m, 1H), 3.70 (m, 4H), 3.57, (m, 1H), 2.63 (broad s, 1H), 2.49 (m, 4H), 2.38 (m, 2), 2.15 (broad s, 1H); ^{13}C NMR ($CDCl_3$, 125 MHz) δ 172.8, 81.3, 76.9, 70.5, 65.7, 52.0, 33.8, 24.0, 15.0.

Bis-TES ether 15

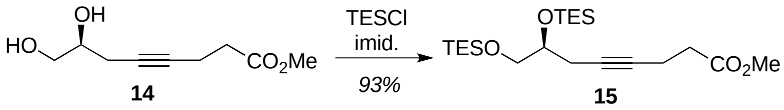

To a solution of diol **14** (328.8 mg, 1.768 mmol) in DMF (10 mL) at 0 °C was added imidazole (484 mg, 7.07 mmol) and chlorotriethylsilane (1.2 mL, 7.07 mmol) followed by Et_3N (1 mL, 7.07 mmol). After 30 min stirring cold hexane/EtOAc (9:1) and ice-cold water were added and the layers were separated and the aqueous layer was extracted with Et_2O. The combined organic layers were washed with water, brine, dried ($MgSO_4$) and the solvent was removed under reduced pressure. Flash chromatography of the residue using 5% EtOAc/petrol as eluent afforded the bis-TES ether **15** as colourless oil (0.683 g, 93%). $[\alpha]_D$ +2.81 (*c* 1.0, CH_2Cl_2); ^{1}H NMR ($CDCl_3$, 400

MHz) δ 3.76 (tt, *J*= 5.8, 5.8 Hz, 1H), 3.69 (s, 3H), 3.53 (m, 2H), 2.50 (m, 4H), 2.42 (m, 1H), 2.24 (m, 1H), 0.95 (tt, *J*= 7.7, 3.7 Hz, 18H), 0.60 (m, 12H); ^{13}C NMR ($CDCl_3$, 100 MHz) δ 172.7, 79.3, 78.2, 72.4, 66.4, 51.9, 33.8, 24.7, 15.0, 7.0, 6.9, 5.1, 4.5. HRMS (ESI) calc for $C_{21}H_{42}O_4Si_2Na$ [M+Na]: 437.2514 found 437.7512.

Alkene 16

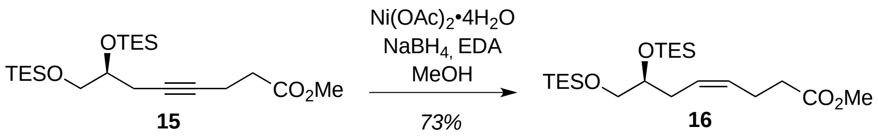

To a degassed suspension of $Ni(OAc)_2$• $4H_2O$ (1.15 g, 4.6 mmol) in MeOH (25mL) under an atmosphere of H_2 was added a solution of $NaBH_4$ (1.0 M in 19:1 MeOH:2.5 M NaOH, 5.2 mL, 52 mmol) and the mixture was stirred until H_2 evolution ceased. EDA (1.44 mL, 21.6 mmol) was added, followed by a solution of alkyne **15** (0.77 g, 1.86 mmol) in MeOH (25 mL) via cannula and the reaction mixture was stirred under H_2 atmosphere at room temperature for 2 h. The solution was diluted with EtOAc and filtered through a pad of Celite. The flitrate was washed with brine, dried ($MgSO_4$) and evaporated to give the crude product. Column chromatography of the residue using 5% EtOAc/petrol as eluent afforded the *Z*-alkene **16** (0.57 g, 73%) as colourless oil. $[\alpha]_D$ +5.81 (*c* 0.85, CH_2Cl_2); IR v_{max} (film) 2954, 1742, 1238, 1095, 1003, 722 cm^{-1}; ^1H NMR ($CDCl_3$, 400 MHz) δ 5.49 (m, 2H), 3.71 (m, 1H), 3.67 (s, 3H), 3.50 (dd, *J* = 9.9, 5.5 Hz, 1H), 3.42 (dd, *J* = 9.9, 6.3 Hz, 1H), 2.37 (m, 5H), 2.18 (dt, *J* = 14.3, 7.0 Hz, 1H), 0.95 (t, *J* = 7.9, 18H), 0.59 (q, *J* = 8.1, 12H); ^{13}C NMR ($CDCl_3$, 125 MHz) δ 173.8, 129.3, 127.5, 73.1, 66.7, 51.64, 34.2, 32.3, 23.1, 7.0, 6.9, 5.1, 4.5; HRMS (ESI) calc for $C_{21}H_{44}O_4Si2Na$ [M+Na]: 439.2670 found 439.2671.

(*E*)-Enyne 17

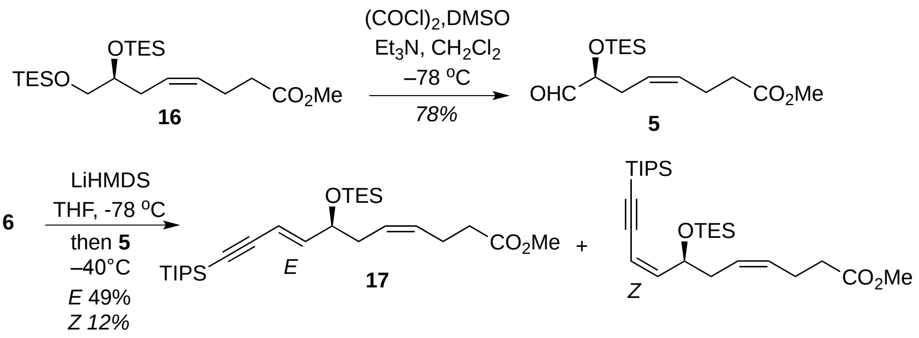

To a solution of oxalyl chloride (0.27 mL, 3.2 mmol) in CH_2Cl_2 (7 mL) at −78 °C was added DMSO (0.5 mL, 7 mmol) and the mixture stirred at −78 °C for 15 min. A solution of alkene **16** (0.34 g, 0.8 mmol) in CH_2Cl_2 (2 mL) was cannulated and the solution stirred at −78 °C for a further 30 min and then at -40°C for an another 30 min. Et_3N (1.5 mL, 10 mmol) was added dropwise and the reaction stirred at −78 °C for 45 min and at room temperature for one hour. The reaction was quenched with water. The layers were separated and the aqueous layer was extracted with DCM. The combined organic layers were washed with water, brine, dried ($MgSO_4$) and the organic solvent was removed under reduced pressure to give the crude aldehyde **5** (187 mg, 0.624 mmol, 78%) as yellow oil, which was used without further purification. To a suspension of the phosphonium bromide **6** (295.7 mg, 0.55 mmol) in dry THF (4 mL) at −78 °C was added LiHMDS (0.9 M in THF, 0.48 mL, 0.44 mmol) dropwise. A solution of the aldehyde **5** (66.5 mg, 0.22 mmol) in dry THF (1.8 mL) was then added to the ylide and the mixture was warmed to −40 °C for 30 min. The reaction was quenched with saturated aqueous NH_4Cl, extracted with Et_2O, washed with H_2O, then brine), dried ($MgSO_4$) then concentrated under reduced pressure to afford the crude product as a yellow oil. It was purified via flash column chromatography using 5% EtOAc/Petrol as the eluent gave the (*E*)-dienyne **17** (51.3 mg, 49%): $[\alpha]_D$ −5.71, (*c* 0.48, CH_2Cl_2); IR ν_{max} (film) 2945, 1743, 1239, 1162, 1074, 1005, 746, 676 cm^{-1}; ^1H NMR ($CDCl_3$, 400 MHz) δ 6.16 (dd, *J* = 6.0, 16.0 Hz, 1H), 5.70 (dd, *J* = 1.6, 16 Hz, 1H), 5.45 (m, 2H), 4.18 (m, 1H), 3.66 (s, 3H), 2.30 (m, 6H), 1.08 (s, 21H), 0.95 (t, *J* = 8.0 Hz, 9H), 0.59 (q, *J* = 8.0 Hz, 6H). ^{13}C NMR ($CDCl_3$, 125 MHz) δ 173.7, 146.6, 130.0, 126.4, 109.6, 105.5,

91.0, 72.4, 51.7, 36.0, 34.1, 23.2, 18.8, 11.4, 7.0, 5.0; HRMS (ESI) calc for $C_{27}H_{50}O_3Si_2Na$ [M+Na]: 501.3191, found 501.3192.

Further elution gave the (*Z*)-enyne (12.8 mg, 12%): $[\alpha]_D$ +6.75 (*c* 0.67, CH_2Cl_2); ^1H NMR ($CDCl_3$, 400 MHz) δ 5.88 (dd, J = 8.8, 11.2 Hz, 1H), 5.51 (dd, *J* = 0.8, 10.8 Hz, 1H), 5.45 (m, 2H), 4.74 (m, 1H), 3.67 (s, 3H), 2.30 (m, 6H), 1.08 (s, 21H), 0.93 (t, *J* = 8.0 Hz, 9H), 0.60 (q, *J* = 8.0 Hz, 6H); ^{13}C NMR ($CDCl_3$, 125 MHz) δ 173.7, 147.1, 129.5, 126.6, 108.9, 103.0, 96.5, 70.6, 51.6, 35.8, 34.2, 23.2, 18.8, 11.4, 6.9, 4.9; HRMS (ESI) calc for $C_{27}H_{50}O_3Si_2Na$ [M+Na]: 501.3191, found 501.3188.

Enyne 3

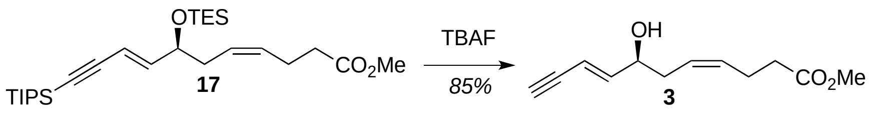

To a solution of enyne **17** (22 mg, 46 μmol) in THF (1mL) at rt was added TBAF (31.8 mg, 92 μmol). The solution was stirred for 1 h then quenched with water and the layers were separated and the aqueous layer extracted with CH_2Cl_2. The combined organic layers were washed with water, brine, dried ($MgSO_4$) and the solvent was removed. Purification by flash chromatography using 5% EtOAc/petrol as eluent afforded the enyne **3** as yellow oil (8.1 mg, 85%). $[\alpha]_D$ −34.5 (*c* 1.4 in CH_2Cl_2); IR ν_{max} (film) 3446, 3289, 1730, 1438, 1162, 960, 749 cm^{-1}; ^1H NMR ($CDCl_3$, 500 MHz) δ 6.25 (dd, *J*= 15.9, 5.3 Hz, 1H), 5.73 (m, 1H), 5.47 (m, 2H), 4.22 (m, 1H), 3.61 (s, 3H), 2.88 (d, *J* = 2.2 Hz, 1H), 2.37 (m, 6H), 1.78 (broad s, 1H); ^{13}C NMR ($CDCl_3$, 125 MHz) δ 173.9, 147.0, 131.6, 125.8, 108.8, 81.8, 78.0, 71.2, 51.8, 35.0, 33.7, 22.8; HRMS (ESI) calc for $C_{12}H_{16}O_3Na$ [M+Na]: 231.0992 found 231.0991.

Alkyne 18

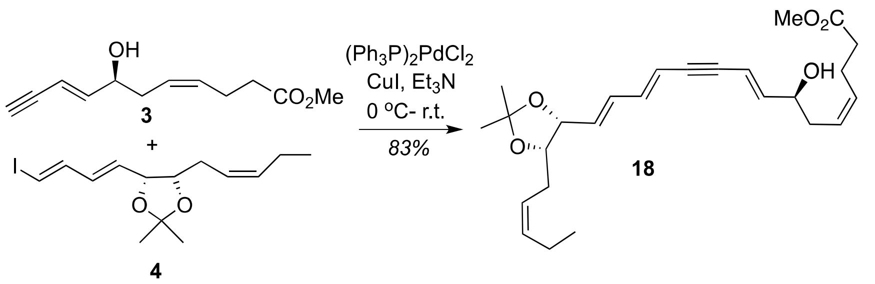

A mixture of the vinyl iodide **4** (113 mg, 0.327 mmol) and dienyne **3** (45.5 mg, 0.218 mmol) was azeotropically dried and then dissolved in MeCN (4 mL) which was degassed twice. To this solution was added $(Ph_3P)_2PdCl_2$ (15.3 mg, 21.8 μmol) and CuI (12.7 mg, 65.4 μmol) and the mixture was degassed once more. The solution was cooled to 0 °C, Et_3N (180μL, 1.3 mmol) then added dropwise and the mixture stirred at 0 °C for 2 h and at room temperature for 17 h. The reaction was quenched with pH 7 buffer solution, the layers were separated and the aqueous layer was extracted with EtOAc. The combined organic layers were washed with water, brine, dried ($MgSO_4$) and the organic solvent was removed under reduced pressure. Column chromatography of the residue using 30% EtOAc/petrol as eluent afforded the alkyne **18** (77.6 mg, 83%) as an orange oil. $[\alpha]_D$ -31.9 (*c* 0.84, CH_2Cl_2); IR ν_{max} (film) 3437, 2958, 2933, 2866, 1736, 1437, 1369, 1244, 1216, 1165, 1101, 1051, 987 cm^{-1}; ^{1}H NMR ($CDCl_3$, 400 MHz) δ 6.58 (dd, *J* = 15.5, 10.9 Hz, 1H), 6.31 (dd, *J* = 15.2, 10.8 Hz, 1H), 6.16 (dd, *J* = 15.9, 5.5 Hz, 1H), 5.90 (dt, *J* = 15.9, 1.9 Hz, 1H), 5.75 (m, 2H), 5.48 (m, 3H), 5.30 (m, 1H), 4.58 (m, 1H), 4.23 (m, 2H), 3.66 (s, 3H), 1.95-2.45 (m, 10H), 1.50 (s, 1H), 1.36 (s, 1H), 0.95 (t, *J*= 7.5 Hz, 3H); ^{13}C NMR ($CDCl_3$, 100 MHz) δ 173.8, 147.7, 145.1, 140.6, 134.3, 132.6, 131.6, 125.9, 124.0, 112.3, 110.1, 108.6, 90.9, 89.6, 78.8, 78.6, 71.5, 51.8, 35.2, 33.7, 28.8, 28.3, 25.7, 22.9, 21.0, 14.2. HRMS (ESI) calc for $C_{26}H_{36}O_5Na$ [M+Na]: 451.2455, found 451.2456.

Enyene triol 19

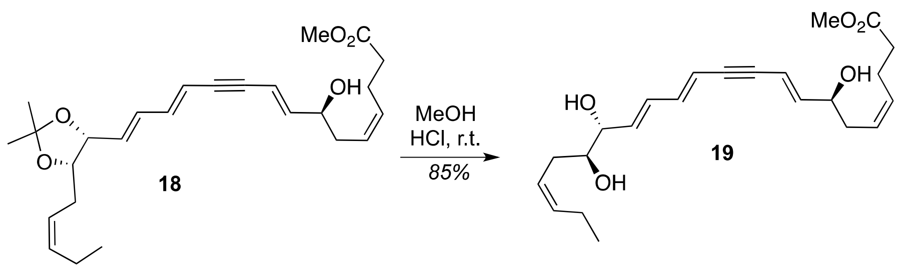

To a solution of alkyne **18** (61.9 mg, 0.144 mmol) in MeOH (2.8 mL) was added HCl (1 M, 0.705 mL, 0.705 mmol) at rt for 3 h. The reaction was quenched with saturated aqueous $NaHCO_3$. The layers were separated and the aqueous layer was extracted with EtOAC. The combined organic layers were washed with water, brine, dried (Na_2SO_4) and the organic solvent was removed under reduced pressure. Column chromatography of the residue using 50% EtOAc/petrol as the eluent afforded the triol **19** (47.4 mg, 85%) as yellow oil. $[\alpha]_D$ −40.5 (*c* 0.562, CH_2Cl_2); IR ν_{max} (film) 3407, 2961, 1745, 1259, 985, 954 cm^{-1}; ^1H NMR ($CDCl_3$, 500 MHz) δ 6.58 (dd, *J*= 15.5, 10.9 Hz, 1H), 6.36 (dd, *J* = 15.2, 10.9 Hz, 1H), 6.16 (dd, *J* = 15.8, 5.5 Hz, 1H), 5.87 (m, 2H), 5.75 (dd, *J* = 15.5, 2.0, 1H), 5.46 (m, 4H), 4.26 (m, 2H), 3.72 (dt, *J*= 8.3, 4.0 Hz, 1H), 3.67 (s, 3H), 2.23 (m, 10H), 0.96 (t, *J*= 7.5 Hz, 3H); ^{13}C NMR ($CDCl_3$, 125 MHz) δ 173.8, 144.8, 140.5, 135.4, 133.2, 131.9, 131.4, 125.7, 123.8, 112.1, 109.9, 90.6, 89.4, 74.6, 73.8, 71.3, 51.6, 35.0, 33.5, 29.9, 22.7, 20.7,14.1; HRMS (ESI) calc for $C_{23}H_{32}O_5Na$ [M+Na]: 411.2142, found 411.2143.

RvD2 methyl ester (20)

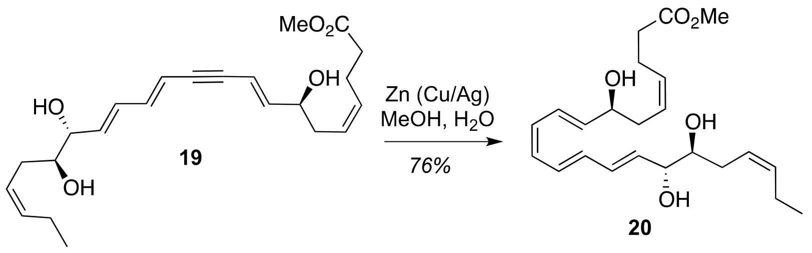

A suspension of Zn dust (5 g) in H_2O (30 mL) was degassed (Ar) for 15 min, and $Cu(OAc)_2$•H_2O (0.5 g) was added. Stirring was continued for 15 min, before $AgNO_3$ (0.5 g) was introduced. The suspension was stirred for another 30 min and was then filtered and washed with H_2O, MeOH, acetone, and Et_2O respectively. The moist Zn mixture was immediately transferred into MeOH/H_2O (1:1,

40 mL). A solution of alkyne **19** (12.3 mg, 29 μmol) in MeOH (10 mL) was added to the suspension of activated Zn (~10 mL) and this was stirred at room temperature for 18 h. The mixture was filtered through a pad of celite, and the filter cake was washed with MeOH. The combined organic extracts were dried (Na_2SO_4) and the solvent was removed under reduced pressure. Purification of the residue by flash chromatography using 70% EtOAc/petrol as the eluent afforded RvD2 methyl ester (**20**) as a yellow solid (8.4 mg, 76%). $[\alpha]_D$ +1.82 (*c* 0.076, CH_2Cl_2); IR ν_{max} (film) 3427, 2958, 1735, 999, 718 cm^{-1}; ^1H NMR ($CDCl_3$, 400 MHz) δ 6.72 (m, 2H), 6.40 (dd, *J* = 15.0, 10.9 Hz, 1H), 6.26 (dd, *J* = 14.6, 10.9 Hz, 1H) 6.02 (m, 2H), 5.80 (m, 2H), 5.47 (m, 4H), 4.27 (m, 1H), 4.23 (m, 1H), 3.71 (m, 1H), 3.67 (s, 3H), 2.24 (m, H10), 0.96 (t, *J* = 7.5 Hz, 3H); ^{13}C NMR ($CDCl_3$, 100 MHz) δ 173.9, 138.0, 135.5, 133.3, 132.9, 131.5, 131.3, 129.6, 128.9, 126.3, 125.6, 124.4, 124.2, 75.1, 74.1, 71.9, 53.6, 35.5, 33.8, 30.1, 23.0, 20.9, 14.4; HRMS (ESI) calc for $C_{23}H_{34}O_5Na$ [M+Na]: 413.2298, found 413.2299.

Resolvin D2 (RvD2) (1)

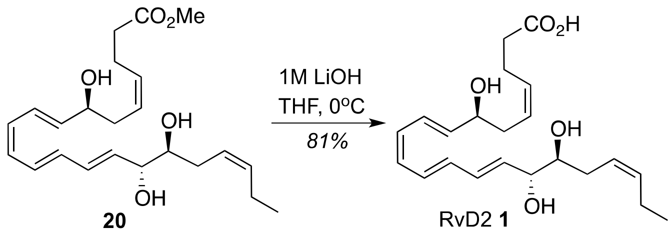

To a solution of the RvD2 methyl ester (**20**) (9.6 mg, 24.6 μmol) in THF (0.5 mL) at 0 °C was added LiOH (1 M, 120 μL, 120 μmol). The solution was stirred at 0 °C for 36 h and diluted with EtOAc and acidified with saturated aqueous NaH_2PO_4 until pH 7. The layers were separated and the aqueous layer was extracted further with EtOAc. The combined organic layers were washed with water, brine, dried (Na_2SO_4) and the solvent was removed under reduced pressure to afford RvD2 (**1**) as yellow oil (7.5 mg, 81%). $[\alpha]_D$ –17.5°(*c* 0.075, CH_2Cl_2). IR ν_{max} (film) 3387, 2917, 2850, 1714, 1408, 1265, 1161, 1052, 865, 792, 737, 704 cm^{-1}; ^1H NMR (CD_3CN, 500 MHz) δ 6.75 (m, 2H), 6.33 (m, 2H), 6.02 (m, 2H), 5.79 (m, 2H), 5.46 (m, 4H), 4.16 (m, 1H), 4.00 (m, 1H), 3.51 (m, 1H),

2.19 (m, H10), 0.94 (t, *J* = 7.5 Hz, 3H); ^{13}C NMR (CD_3CN, 125 MHz) δ 175.1, 139.6, 135.3, 134.8, 134.7, 133.1, 131.4, 130.7, 130.6, 129.3, 128.1, 126.9, 126.3, 76.2, 75.9, 72.8, 36.6, 34.6, 31.8, 24.1, 21.9, 15.0; HRMS (ESI) Calcd for $C_{22}H_{32}O_5Na$ [M+Na]: 399.2142, found: 399.2142.

References

[1] Rodríguez, A. R.; Spur, B. W. *Tetrahedron Lett.* **2012**, *53*, 4169.

[2] Rodríguez, A. R.; Spur, B. W. *Tetrahedron Lett.* **2004**, *45*, 8717.
