# Supplementary material for: Total synthesis of the endogenous inflammation resolving lipid resolvin D2 using a common lynchpin
Source: Beilstein J Org Chem. 2013 Dec 3;9:2762–6. doi: 10.3762/bjoc.9.310 (PMC3869264; doi:10.3762/bjoc.9.310)
Supplement: File 2 — 1H and 13C NMR spectra of all intermediates and the mass spectrum of RvD2 (1). [file Beilstein_J_Org_Chem-09-2762-s002.pdf]

Supporting Information File 2

for

Total synthesis of the endogenous inflammation resolving lipid resolvin D2 using a common lynchpin

John Li[1], May May Leong[1], Alastair Stewart[2] and Mark A. Rizzacasa*[1]

Address: [1]School of Chemistry, The Bio21 Institute, The University of Melbourne, Parkville, Victoria 3010, Australia and [2]Department of Pharmacology and Therapeutics, The University of Melbourne, Parkville, Victoria, 3010, Australia.

Email: Mark A. Rizzacasa* - masr@unimelb.edu.au

* Corresponding author

^{1}H and ^{13}C NMR spectra of all intermediates and the mass spectrum of RvD2 (1)

^{1}H NMR spectrum of alcohol **10** S3

^{13}C NMR spectrum of alcohol **10** S4

^{1}H NMR spectrum of aldehyde **7** S5

^{13}C NMR spectrum of aldehyde **7** S6

^{1}H NMR spectrum of enyne **11** S7

^{13}C NMR spectrum of enyne **11** S8

^{1}H NMR spectrum of *Z*-isomer of **11** S9

^{13}C NMR spectrum of *Z*-isomer of **11** S10

^{1}H NMR spectrum of enyne **12** S11

^{13}C NMR spectrum of enyne **12** S12

^{1}H NMR spectrum of vinyl iodide **4** S13

^{13}C NMR spectrum of vinyl iodide **4** S14

^{1}H NMR spectrum of diol **14** S15

^{13}C NMR spectrum of diol **14** S16

^{1}H NMR spectrum of bis-(*S*)-Mosher ester S17

^{1}H NMR spectrum of bis-TES ether **15** S18

^{13}C NMR spectrum of bis-TES ether **15** S19

^{1}H NMR spectrum of alkene **16** S20

^{13}C NMR spectrum of alkene **16** S21

^{1}H NMR spectrum of aldehdye **5** S22

^{13}C NMR spectrum of aldehdye **5** S23

^{1}H NMR spectrum of enyne **17** S24

^{13}C NMR spectrum of enyne **17** S25

^{1}H NMR spectrum of *Z*-isomer of enyne **17** S26

^{13}C NMR spectrum of *Z*-isomer of enyne **17** S27

^{1}H NMR spectrum of enyne **3** S28

^{13}C NMR spectrum of enyne **3** S29

^{1}H NMR spectrum of alkyne **18** S30

^{13}C NMR spectrum of alkyne **18** S31

^{1}H NMR spectrum of triol **19** S32

^{13}C NMR spectrum of triol **19** S33

^{1}H NMR spectrum of RvD2 methyl ester **20** S34

^{13}C NMR spectrum of RvD2 methyl ester **20** S35

^{1}H NMR spectrum of RvD2 **1** S36

^{13}C NMR spectrum of RvD2 **1** S37

Low resolution mass spectrum of RvD2 **1** (negative ion mode) S38

^{1}H NMR ($CDCl_3$, 400MHz)

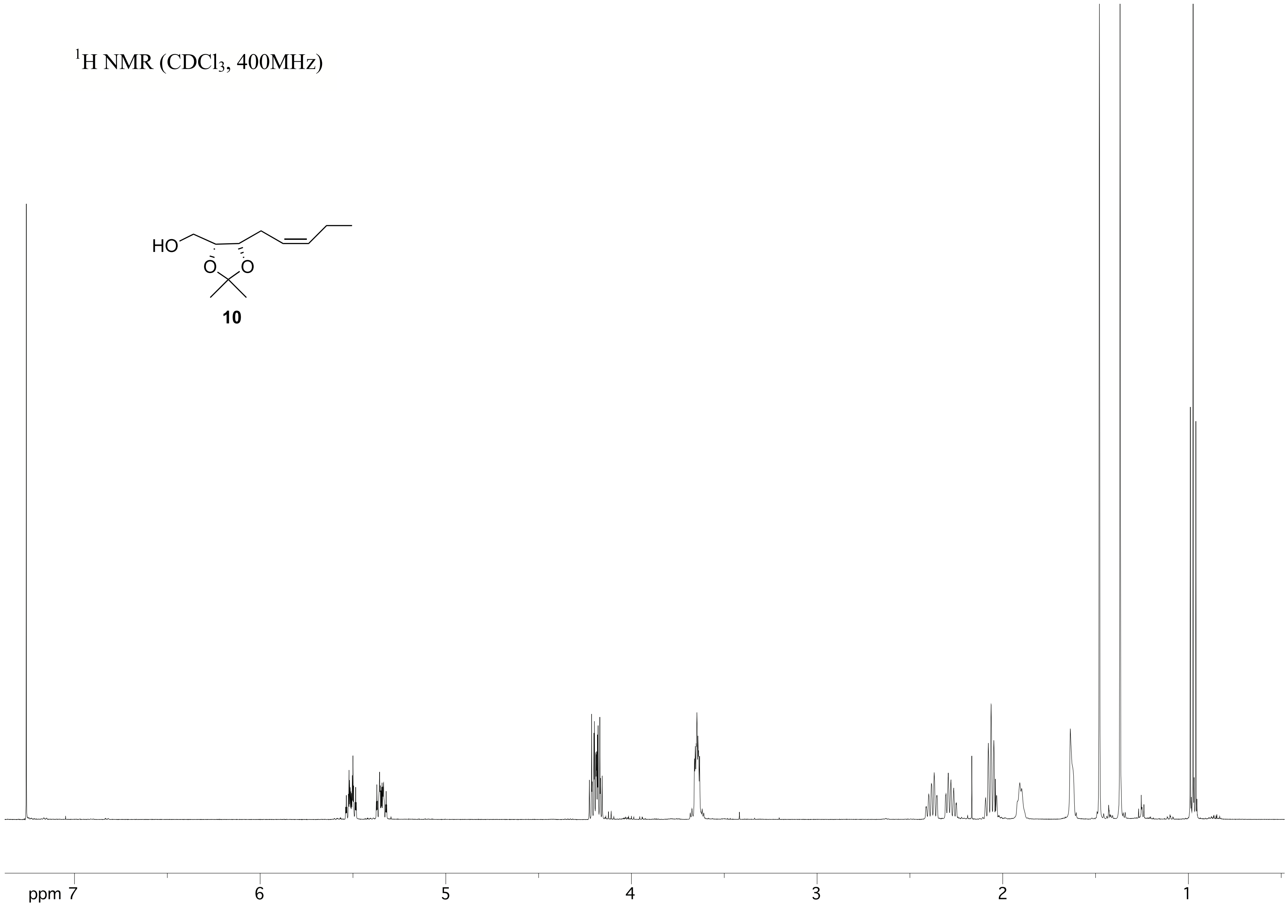

^{13}C NMR ($CDCl_3$, 100MHz)

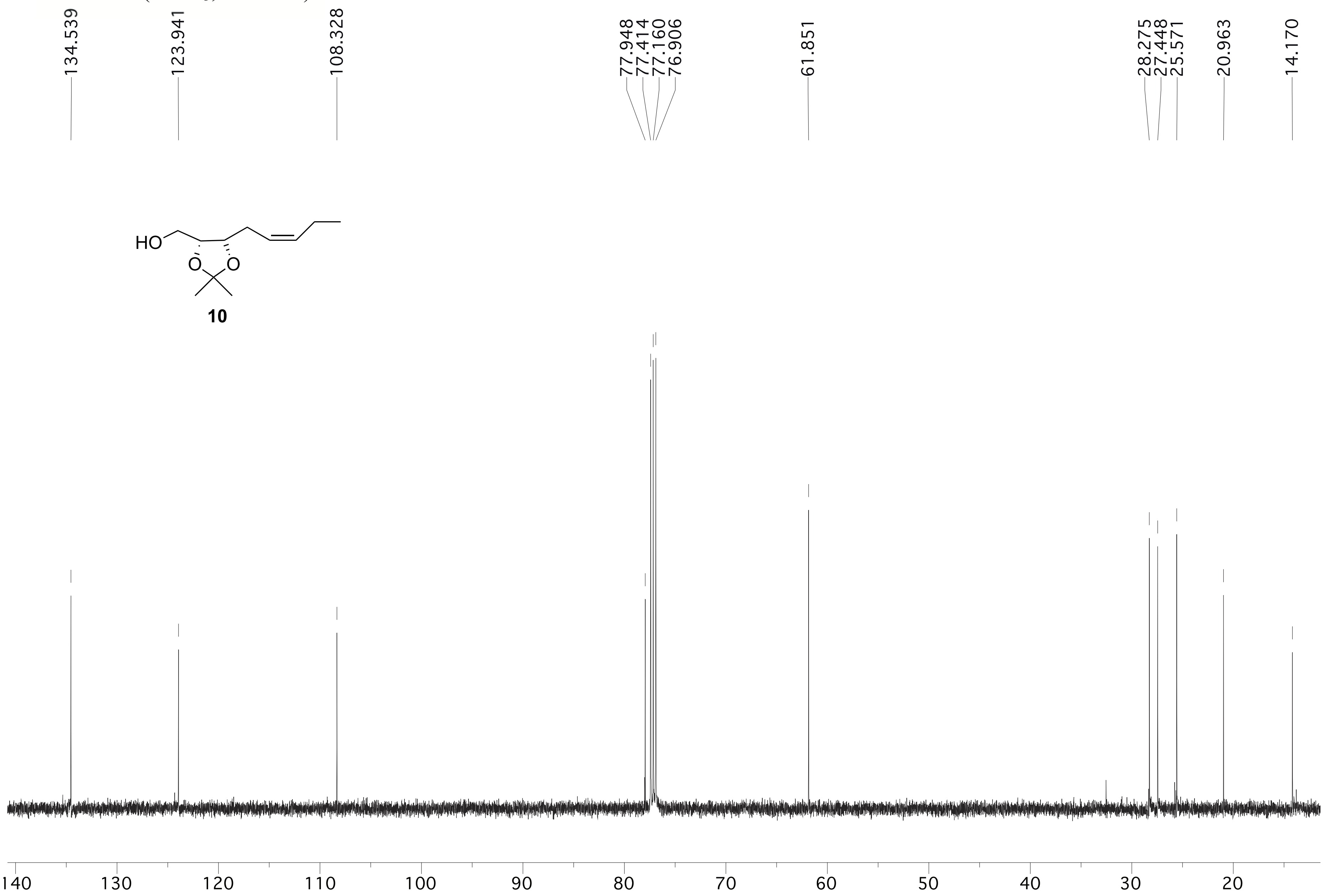

^{1}H NMR ($CDCl_3$, 400MHz)

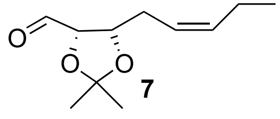

7

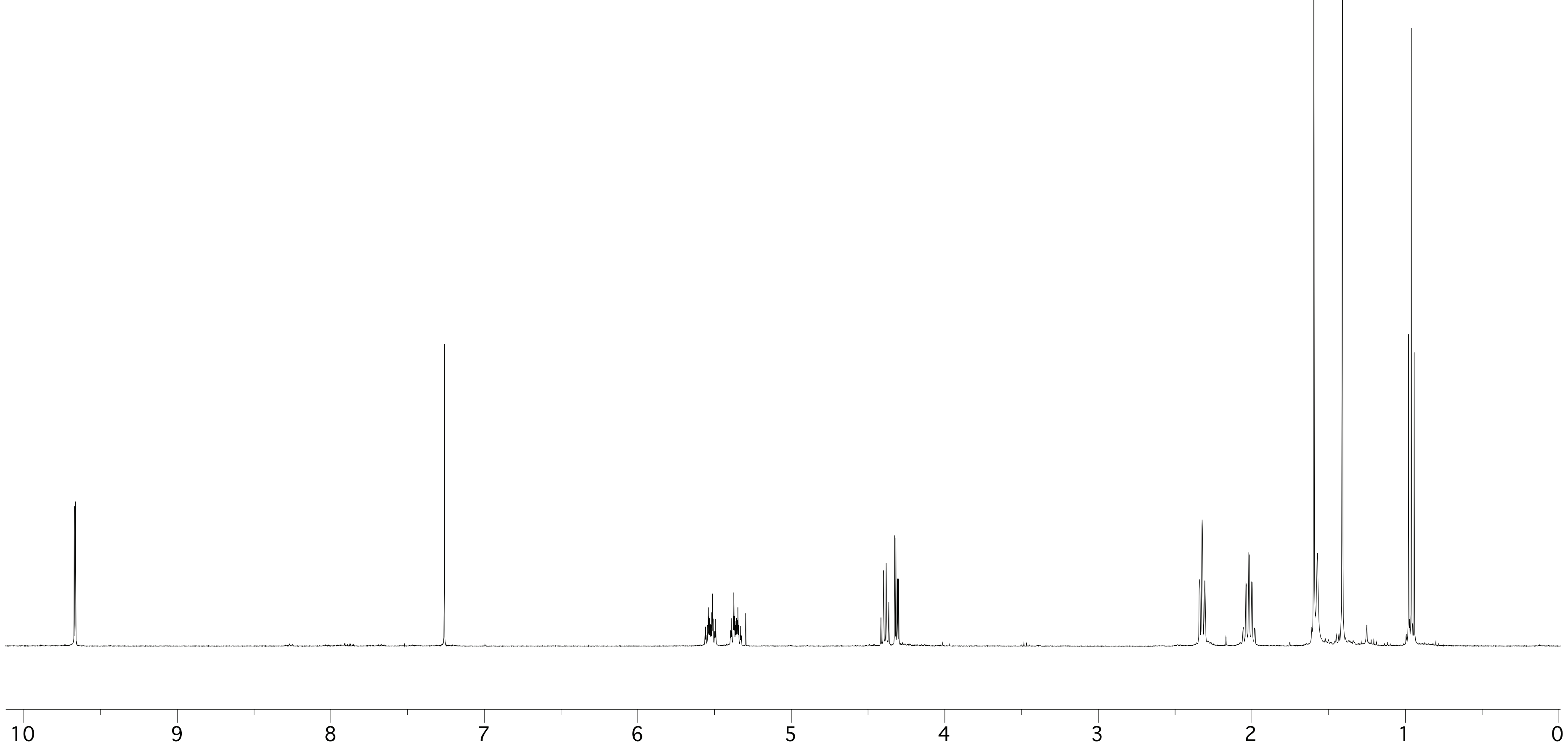

^{13}C NMR ($CDCl_3$, 100MHz)

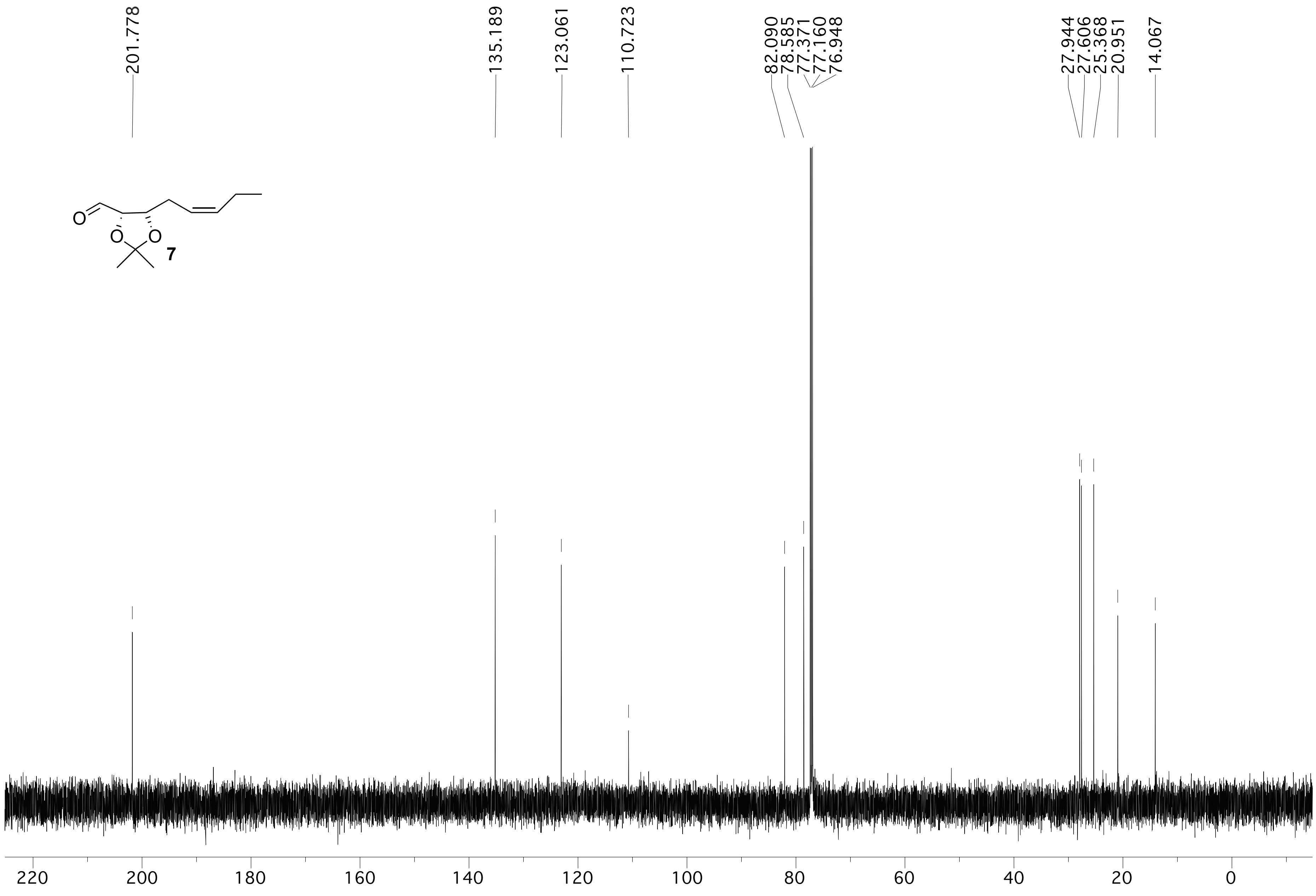

^1H NMR ($CDCl_3$, 600MHz)

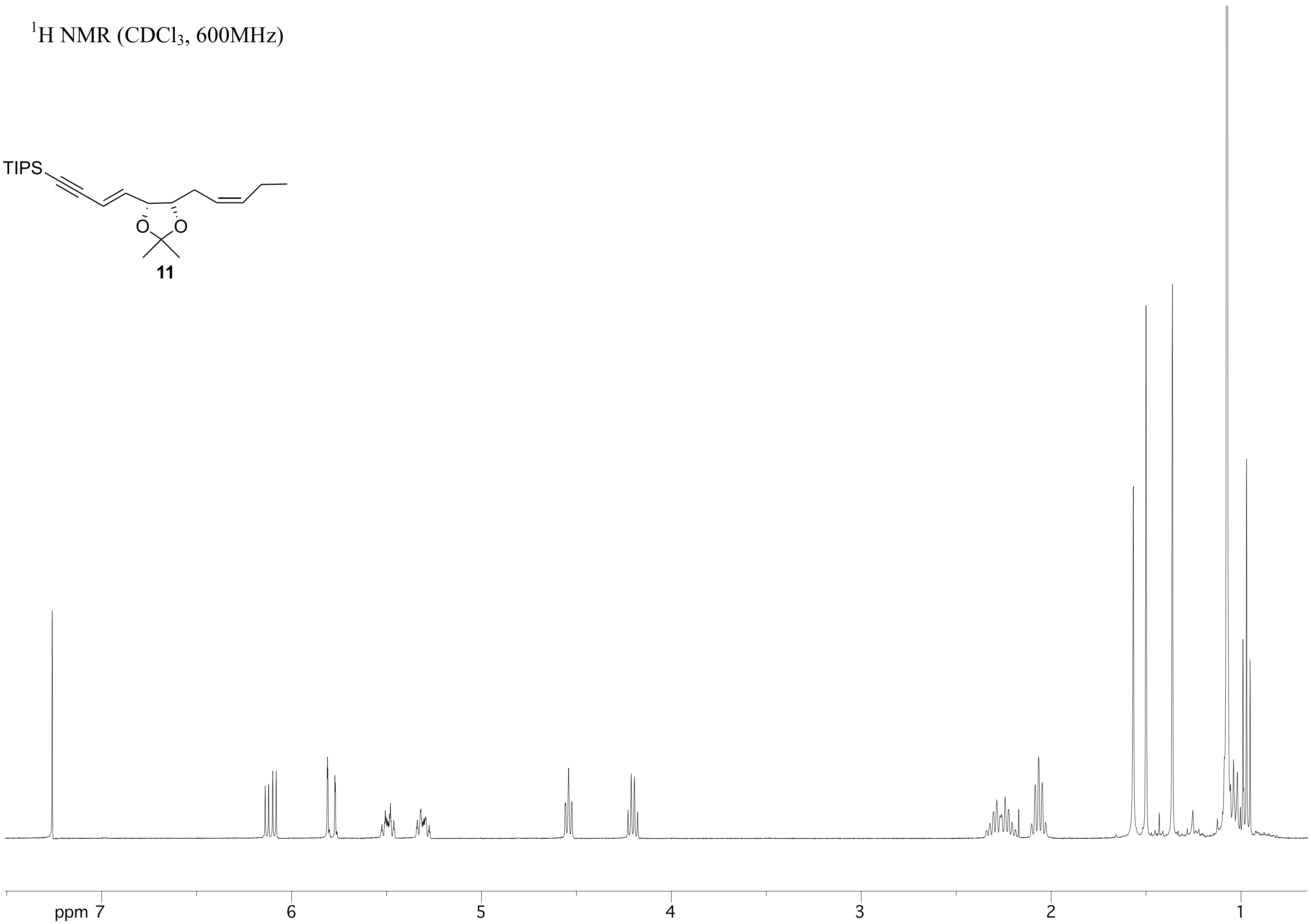

^{13}C NMR (CDCl$_3$, 150MHz)

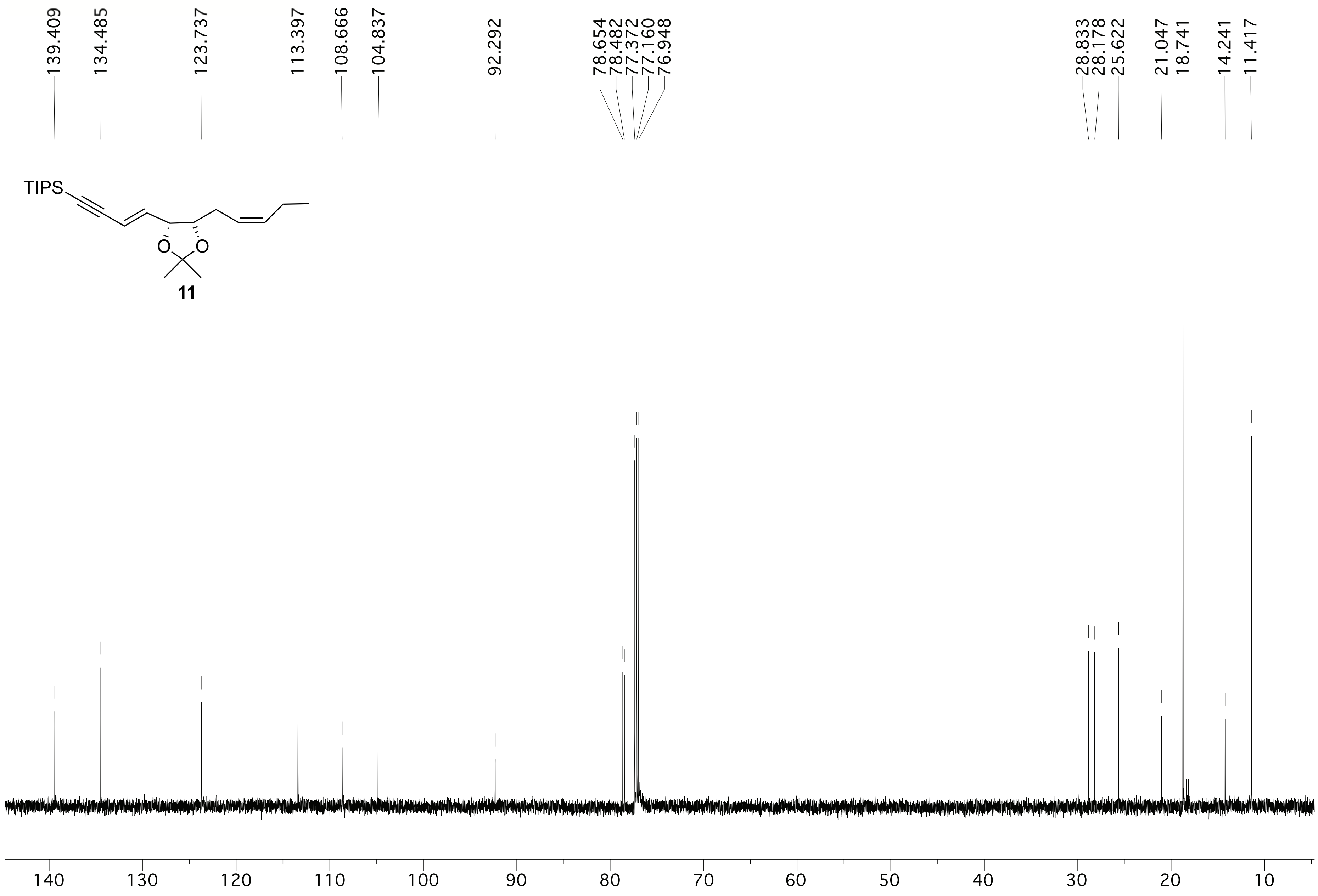

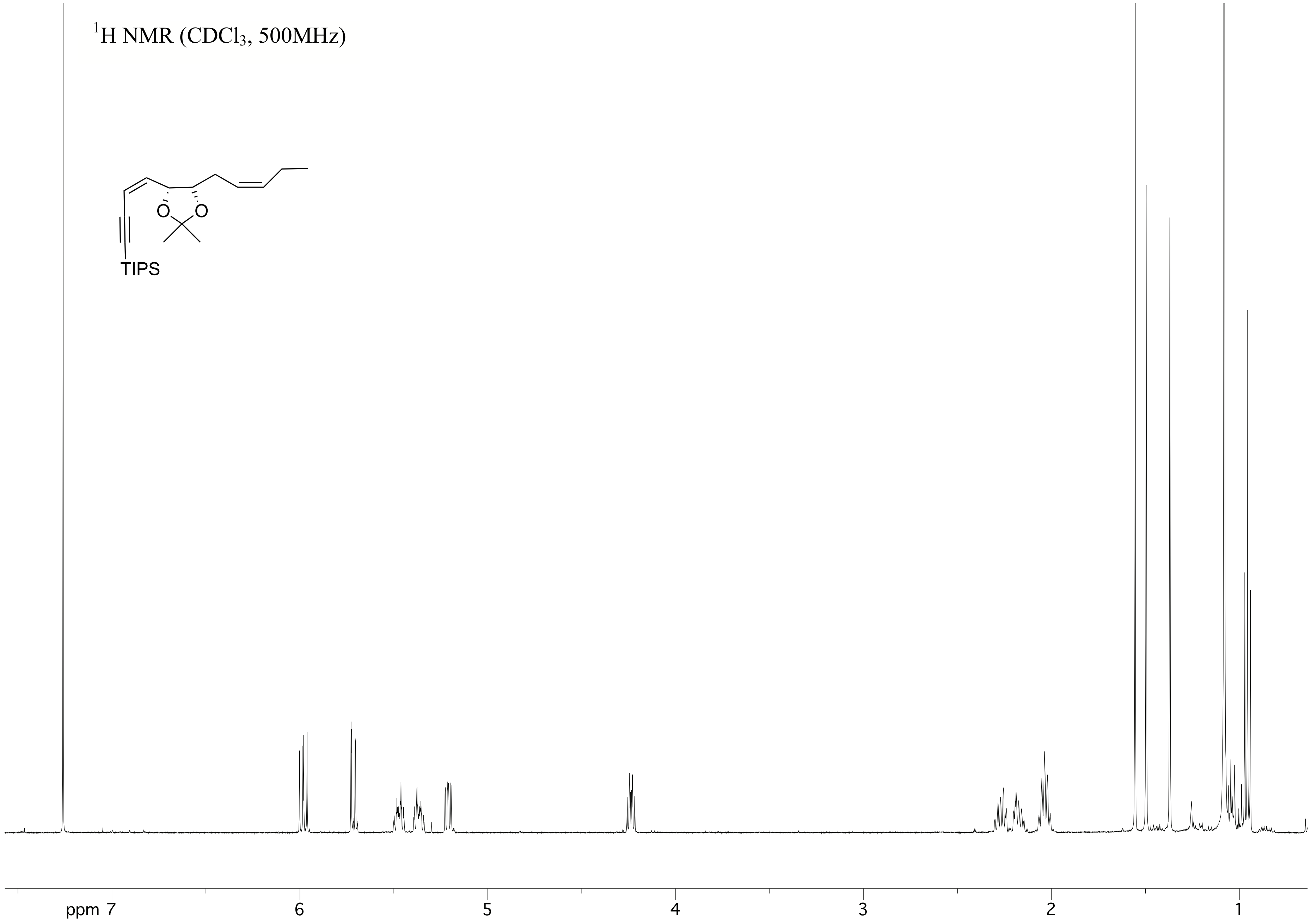

^{1}H NMR ($CDCl_3$, 500MHz)
TIPS
ppm 7
6
5
4
3
2
1

^{13}C NMR ($CDCl_3$, 125MHz)

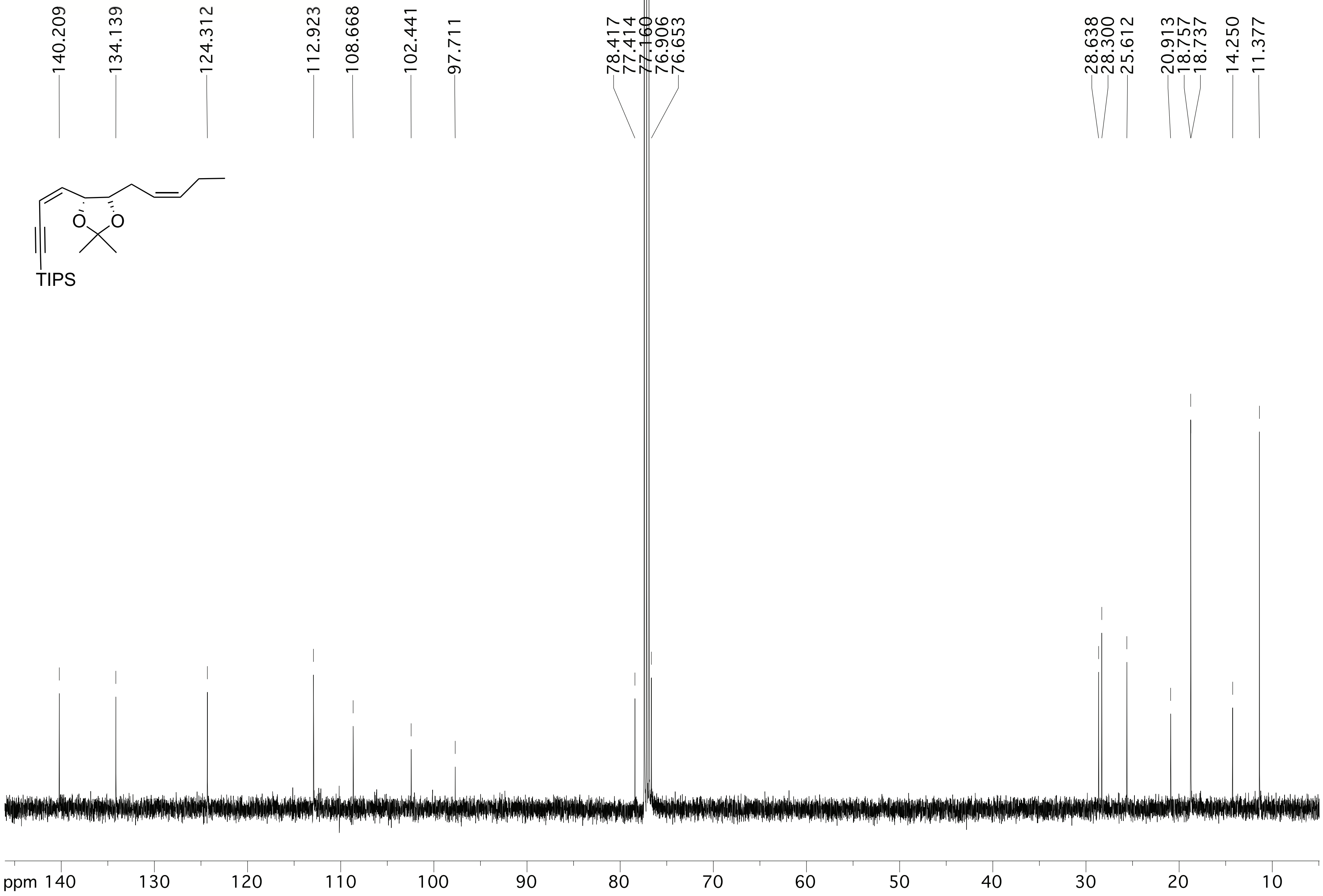

^{1}H NMR ($CDCl_3$, 500MHz)

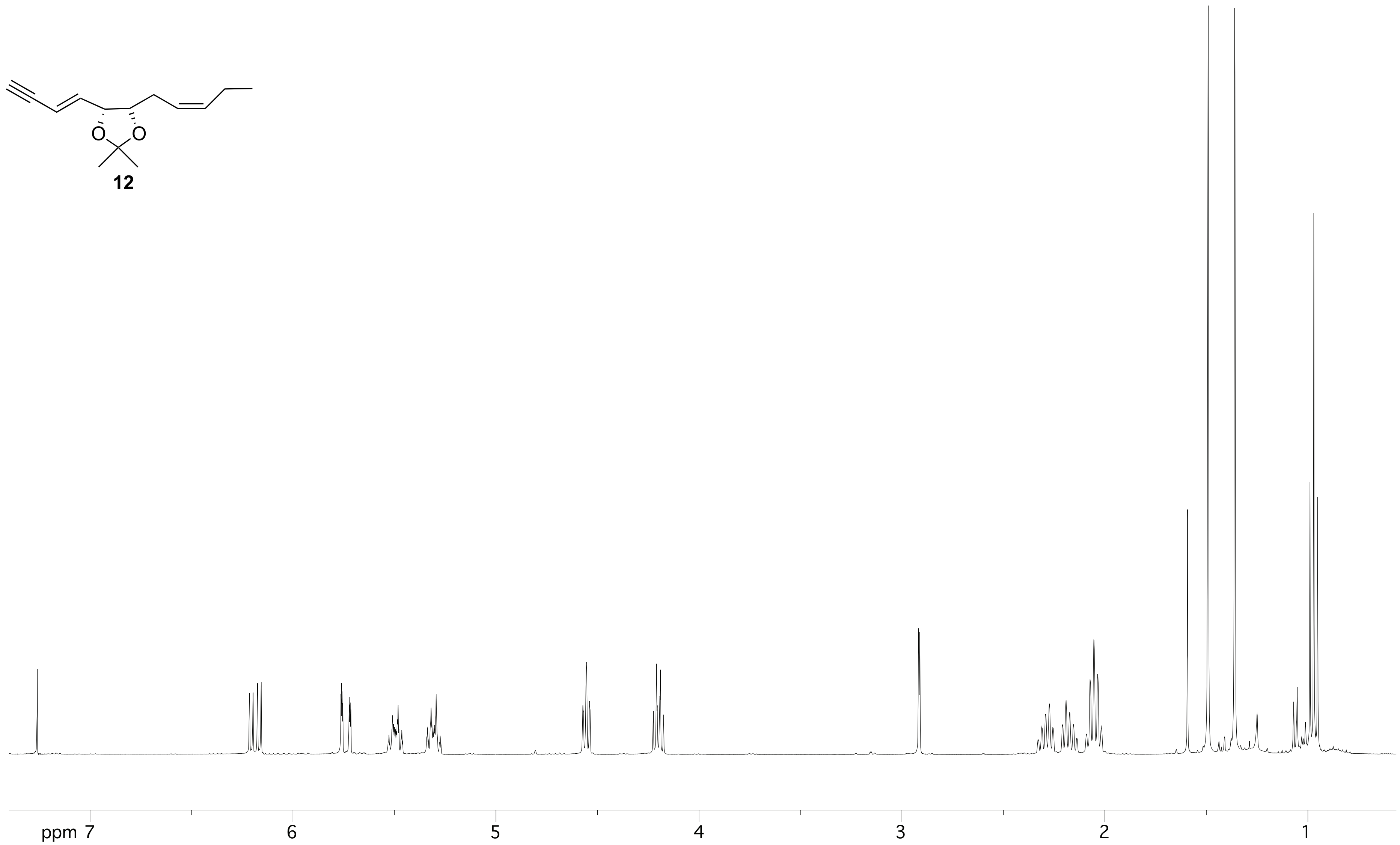

12

^{13}C NMR ($CDCl_3$, 125MHz)

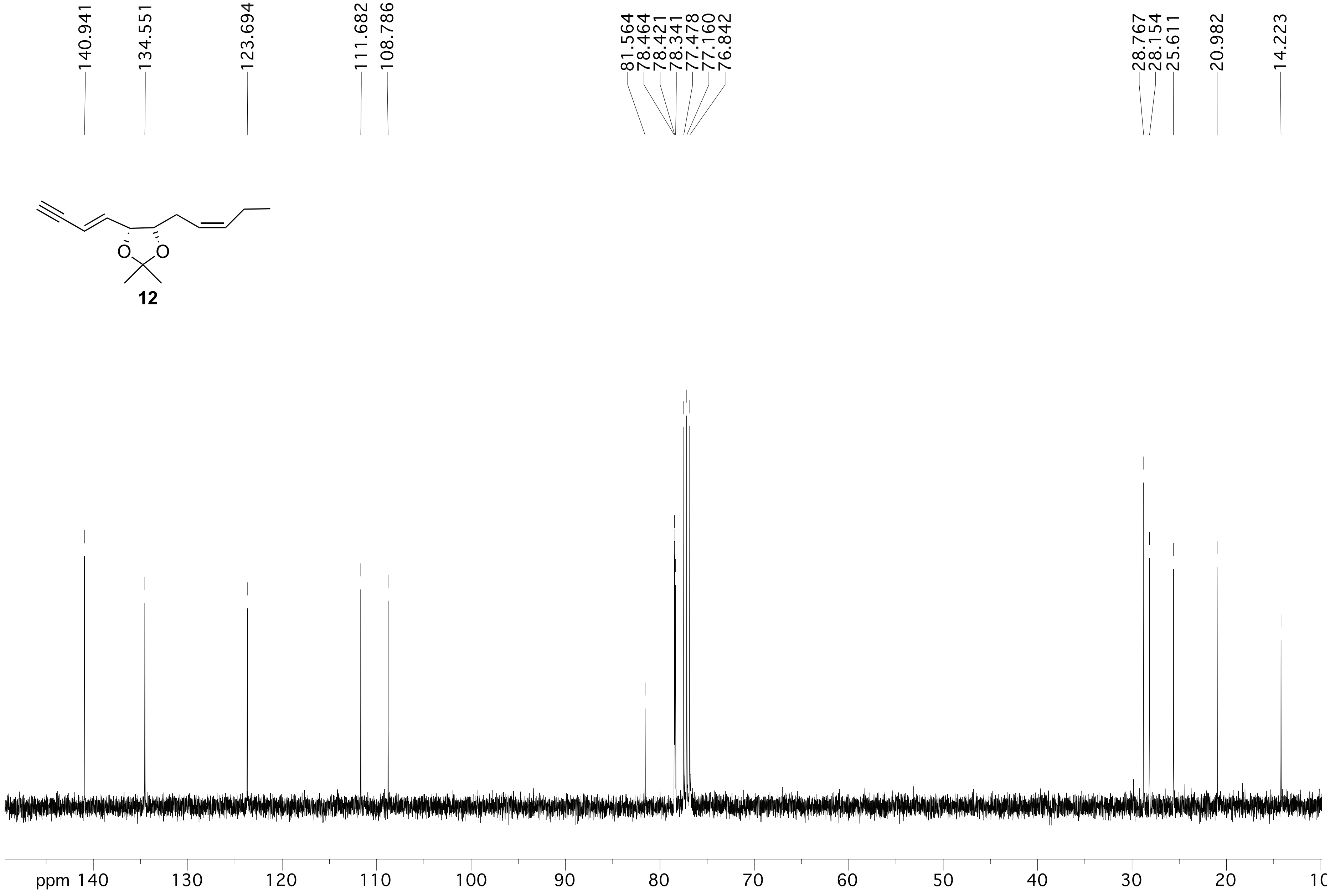

^{1}H NMR ($CDCl_3$, 600MHz)

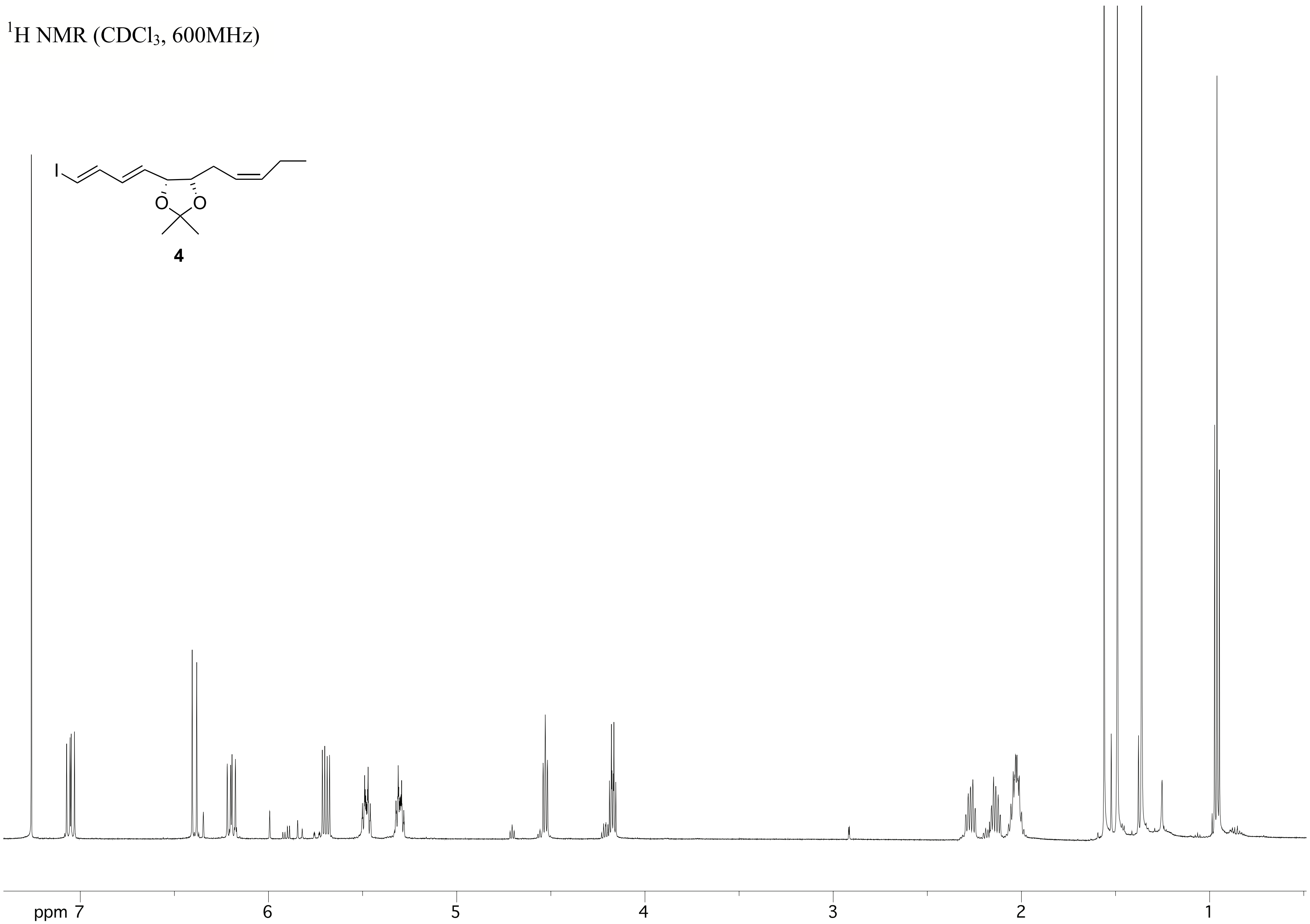

^{13}C NMR ($CDCl_3$, 150MHz)

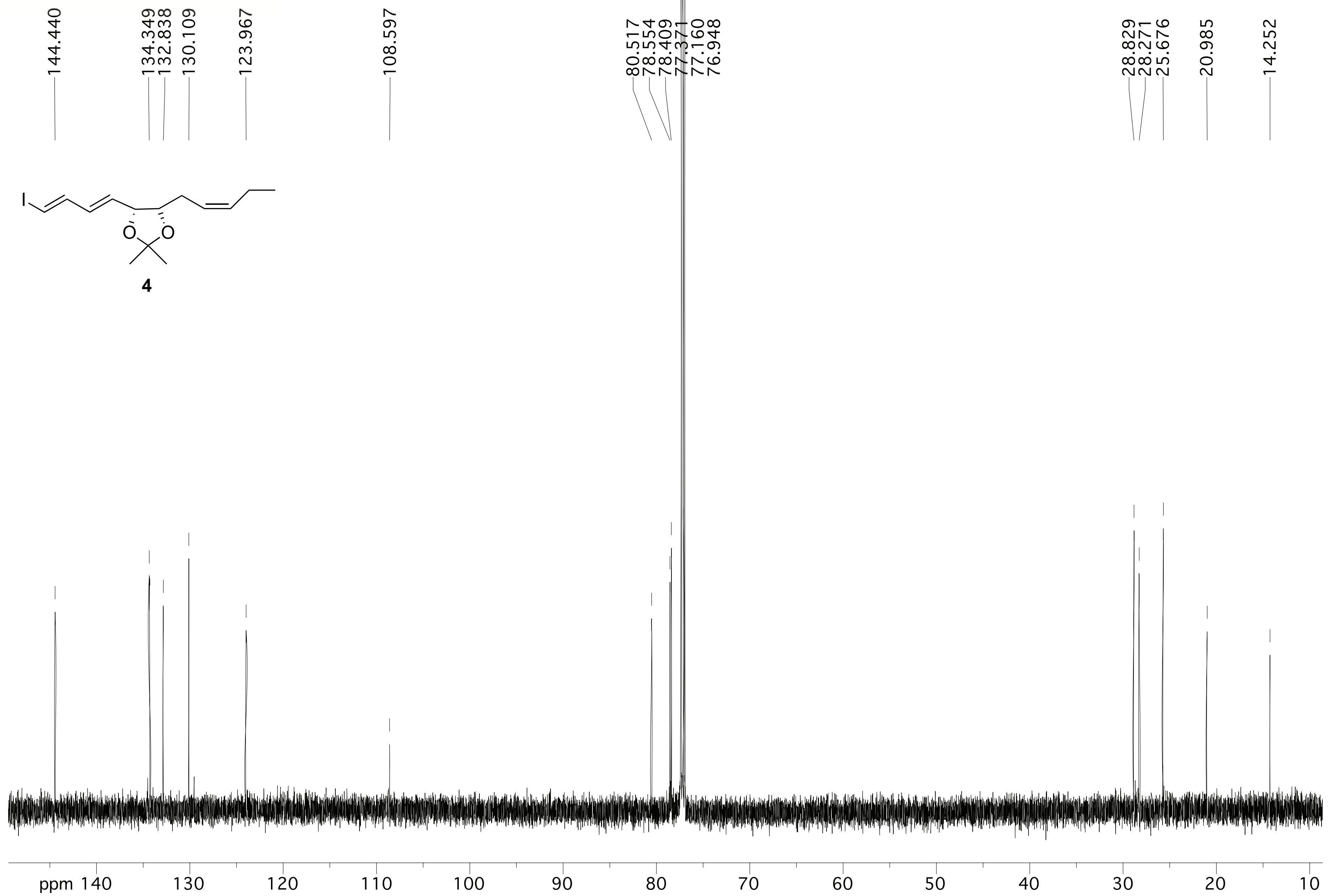

^{1}H NMR ($CDCl_3$, 500MHz)

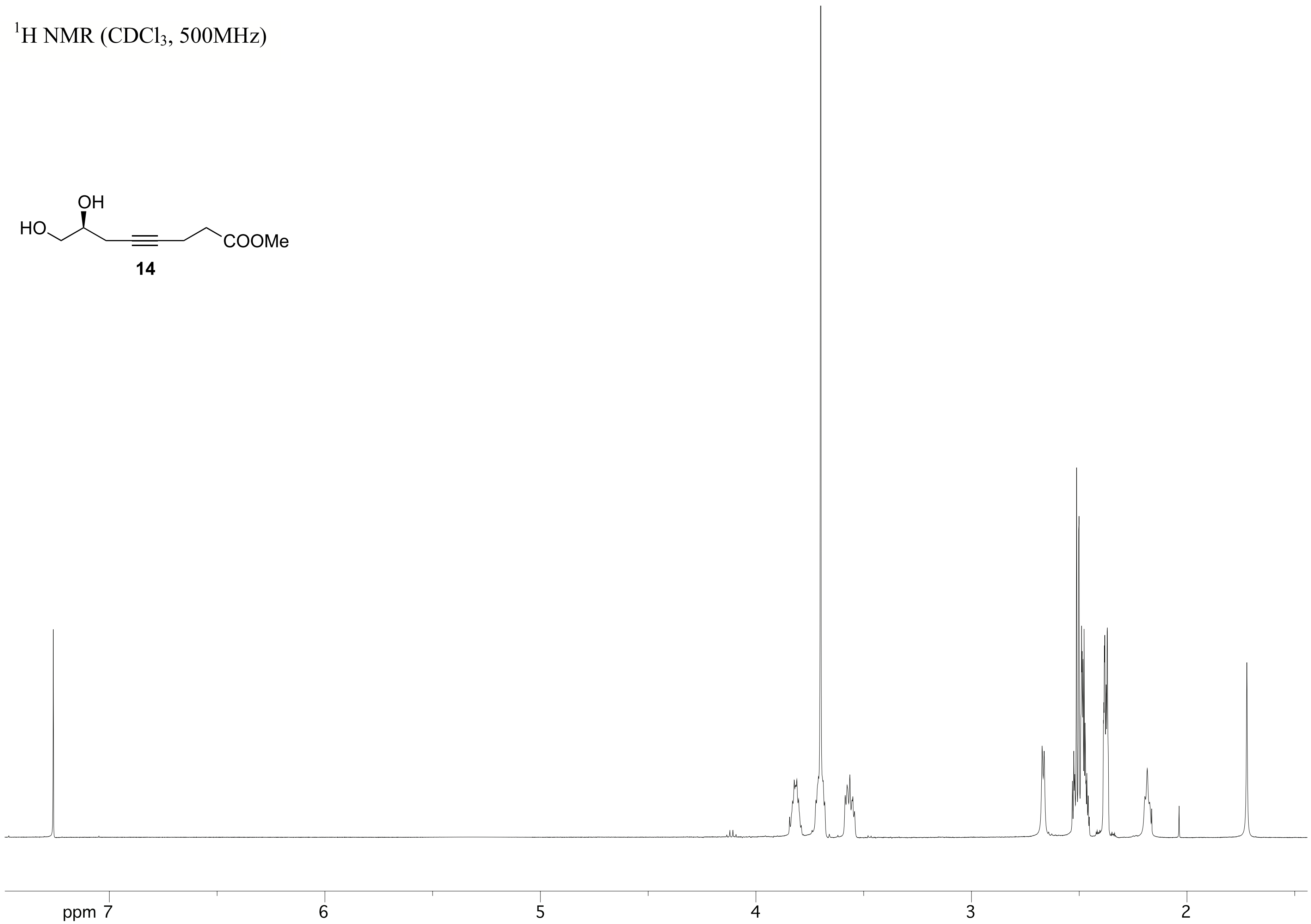

^{13}C NMR ($CDCl_3$, 125MHz)

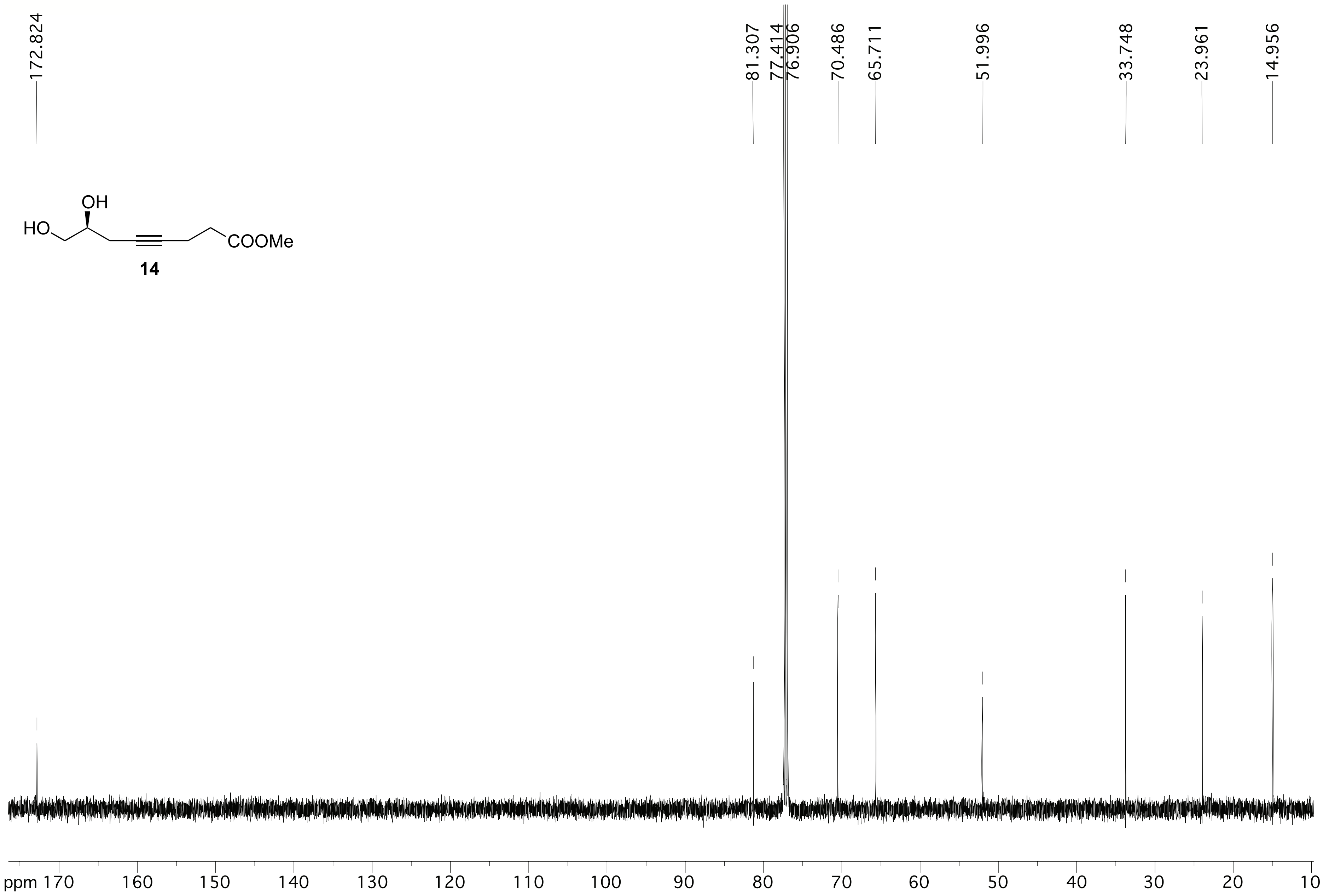

^{1}H NMR ($CDCl_3$, 400MHz)

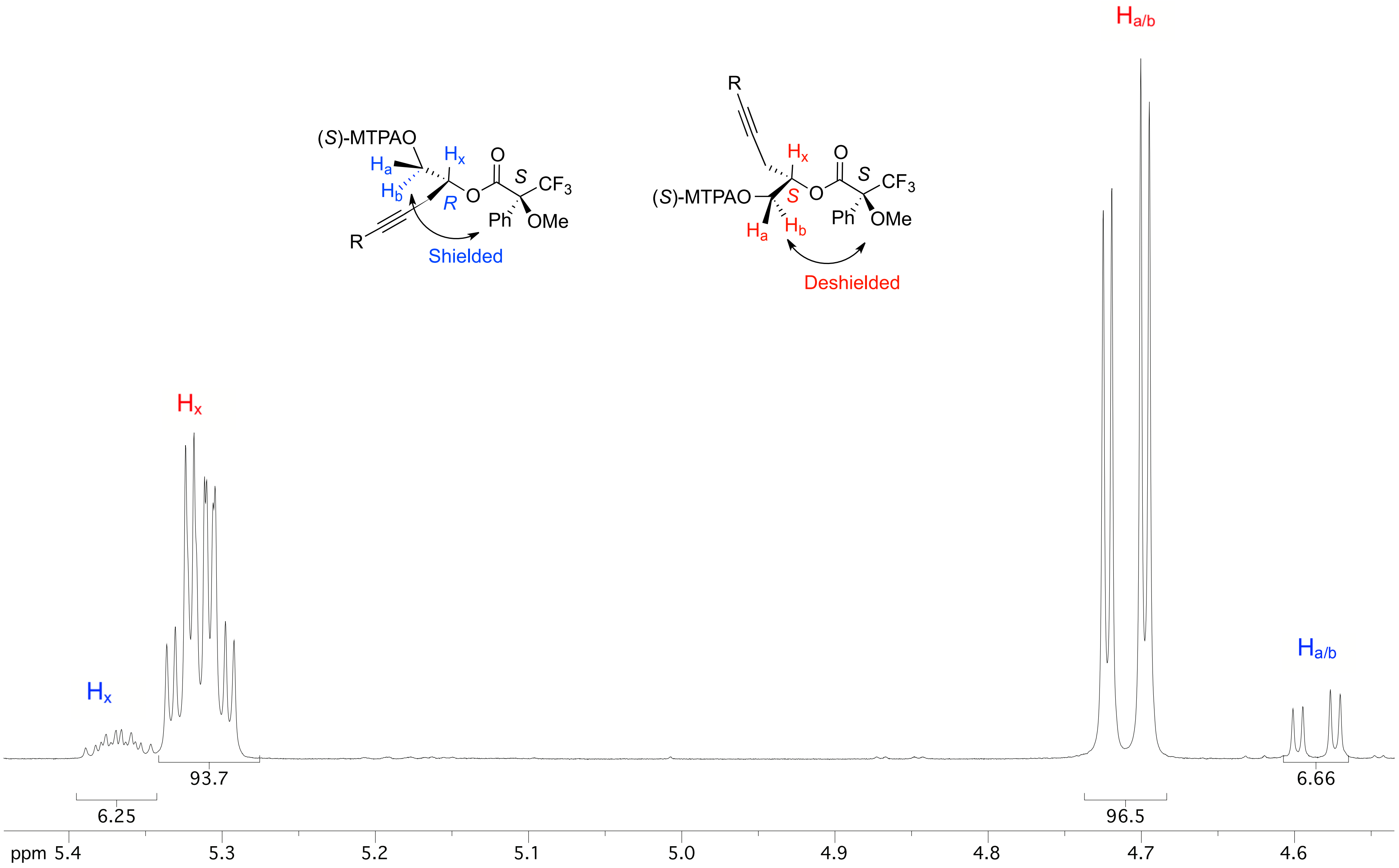

^{1}H NMR ($CDCl_3$, 400MHz)

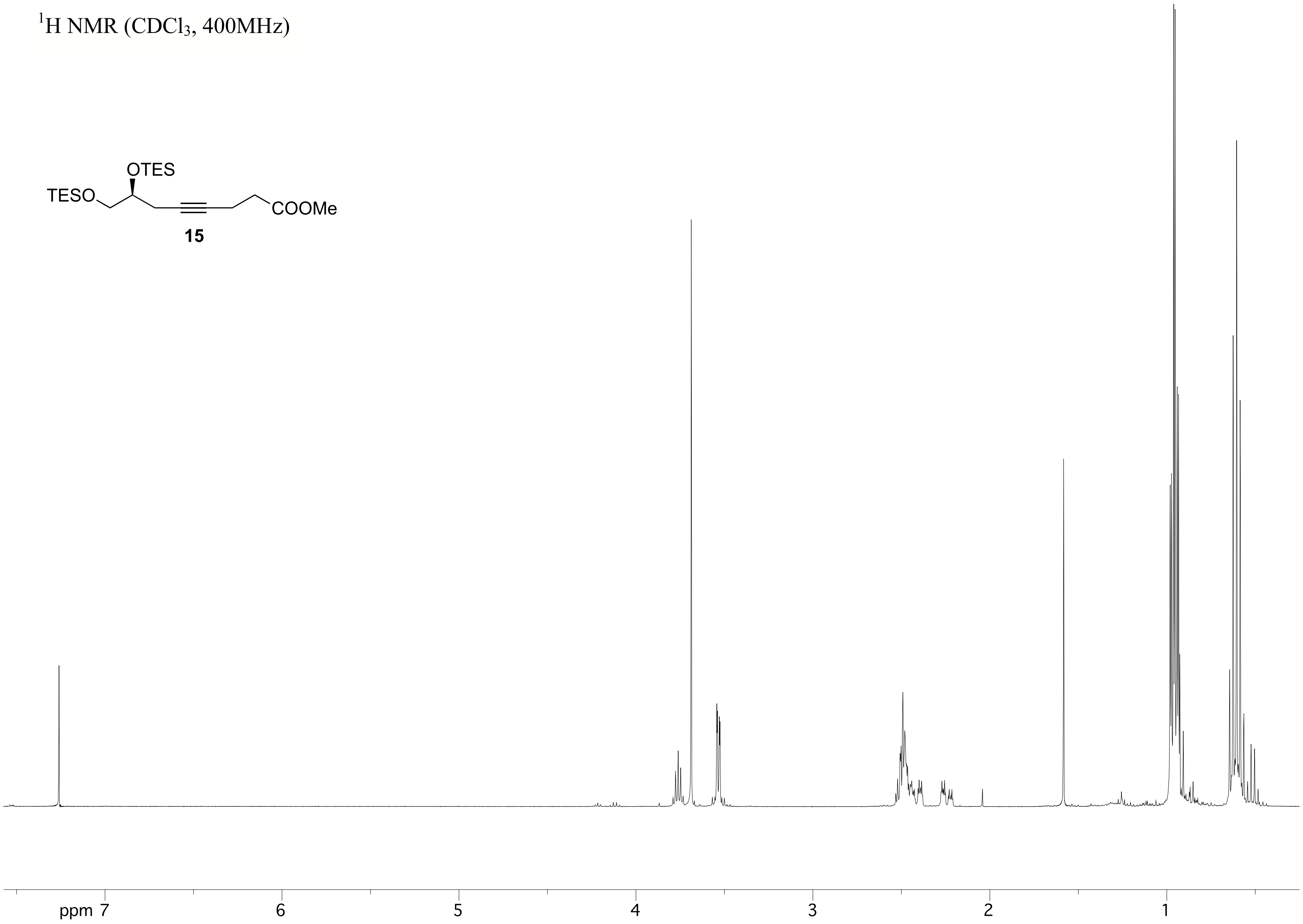

^{13}C NMR ($CDCl_3$, 100MHz)

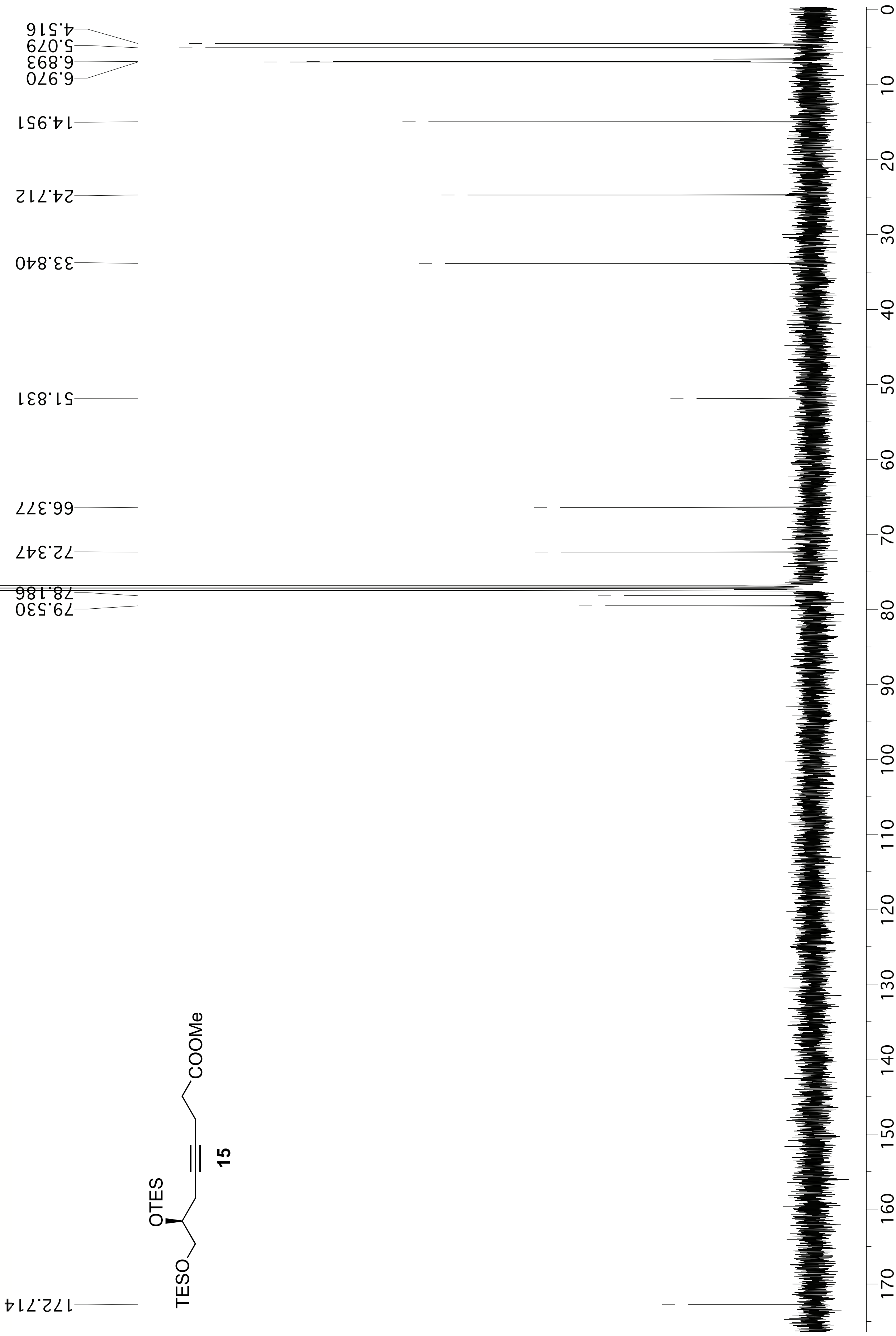

^{1}H NMR ($CDCl_3$, 600MHz)

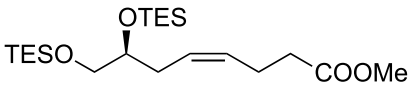

16

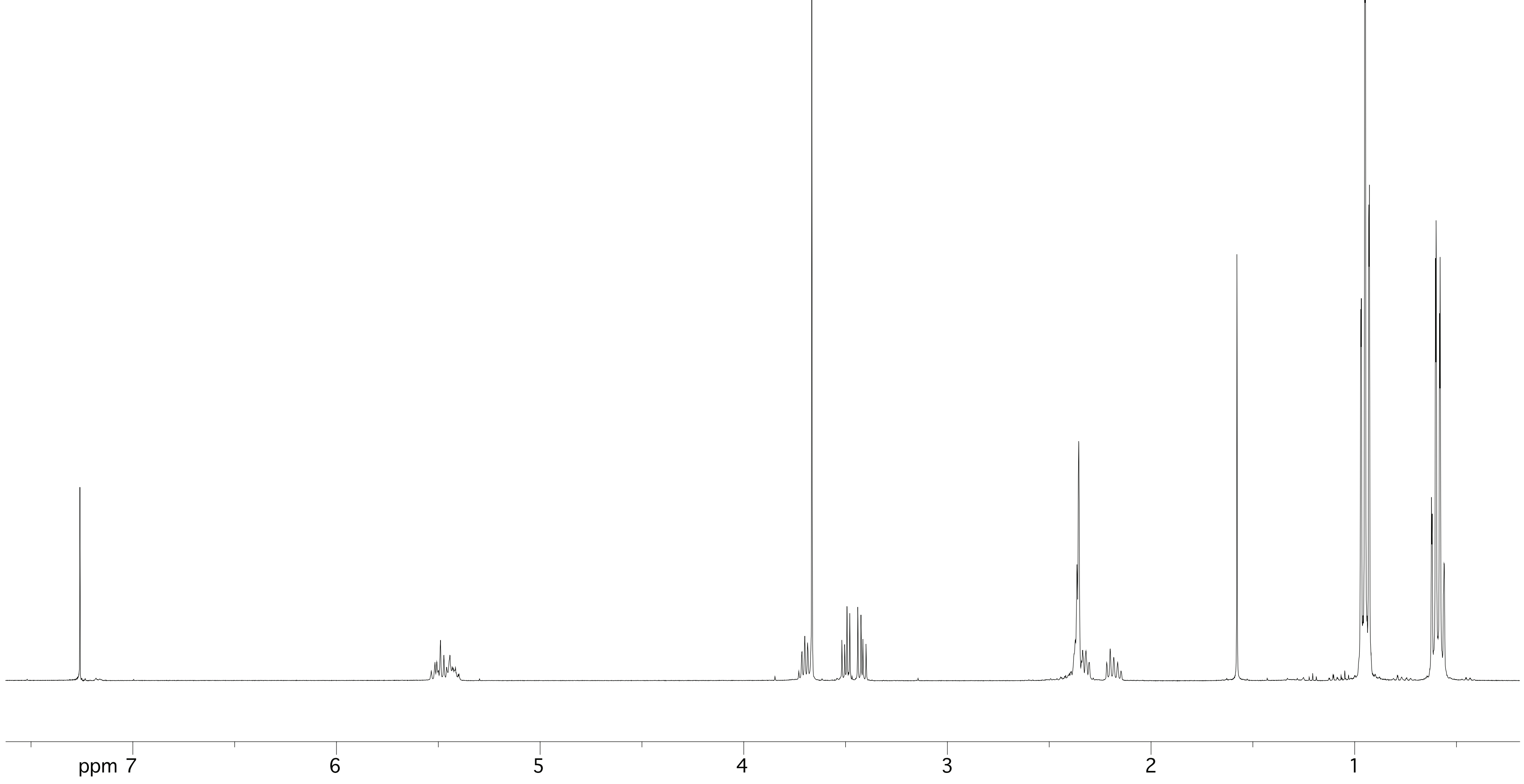

^{13}C NMR ($CDCl_3$, 150MHz)

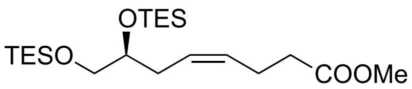

16

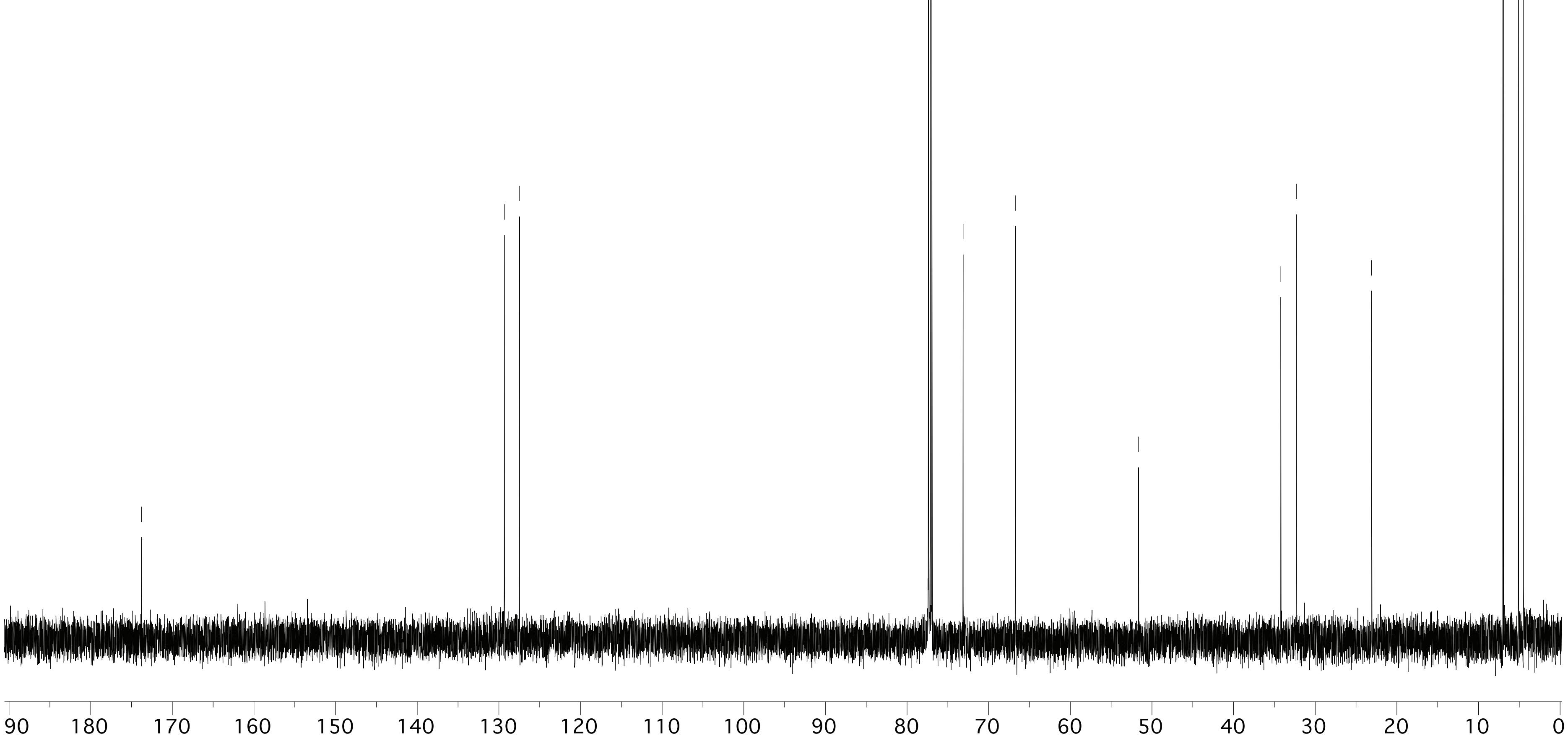

^{1}H NMR ($CDCl_3$, 500MHz)

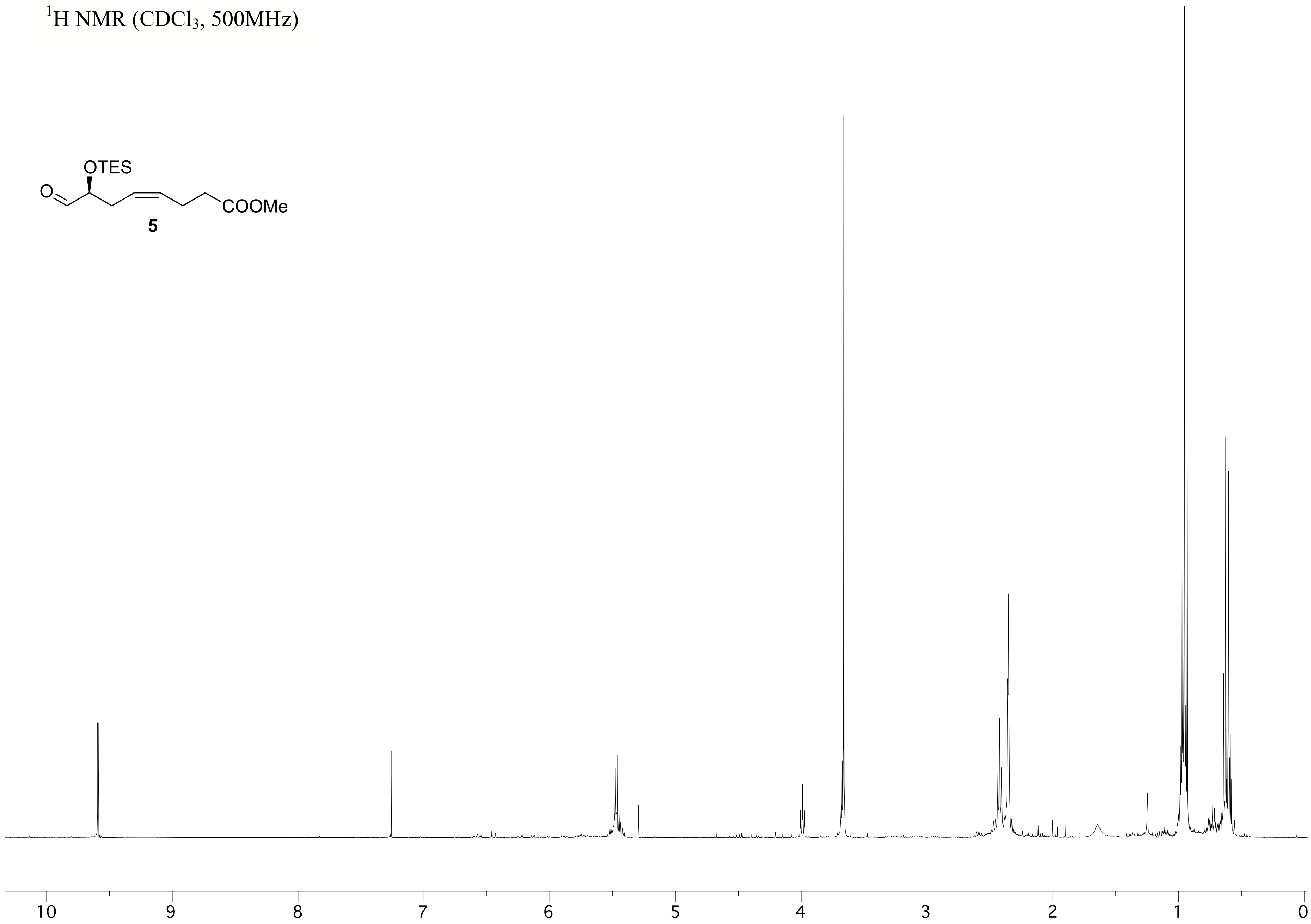

^{13}C NMR ($CDCl_3$, 125MHz)

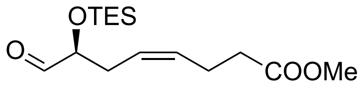

5

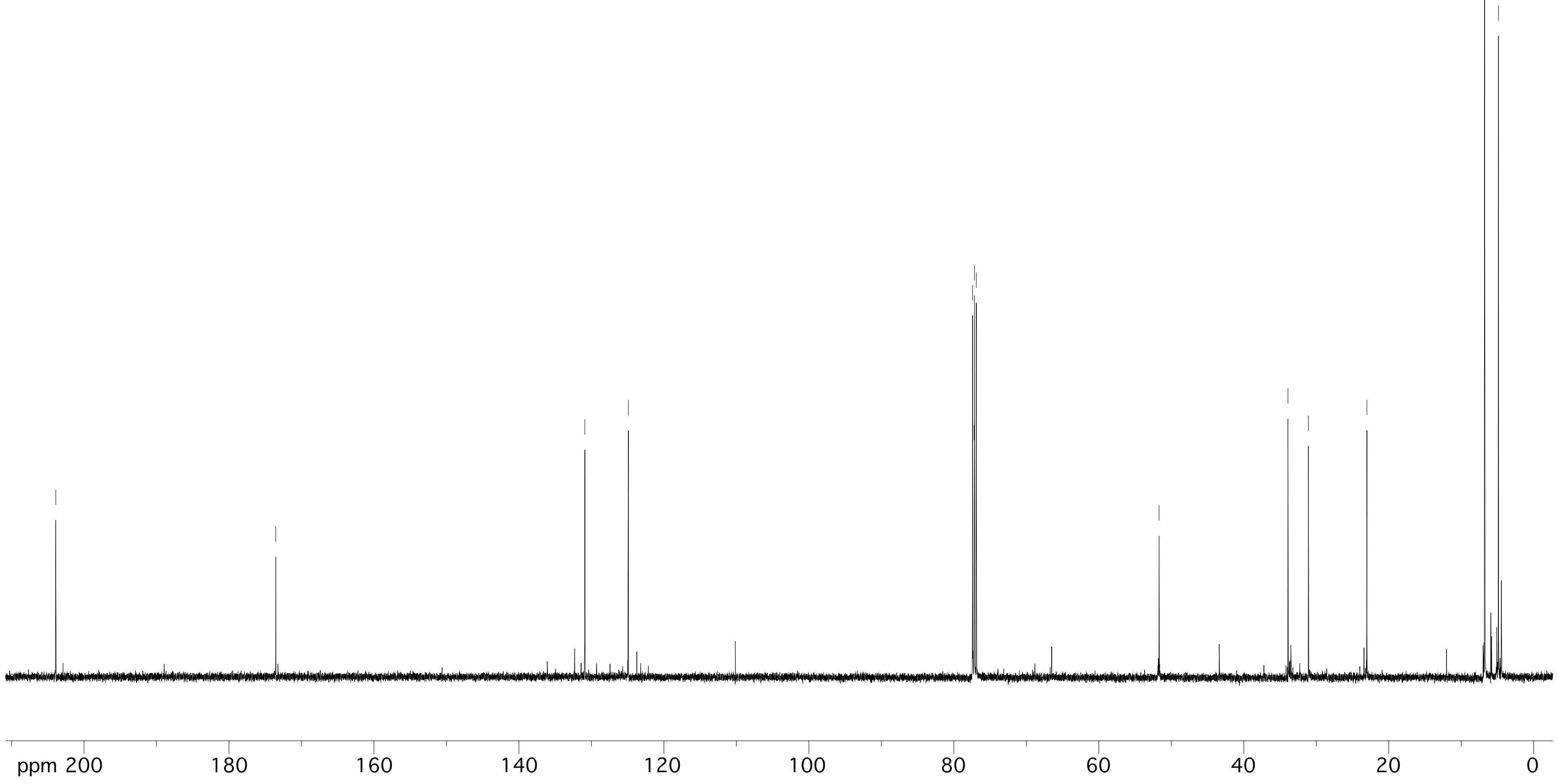

^{1}H NMR ($CDCl_3$, 500MHz)

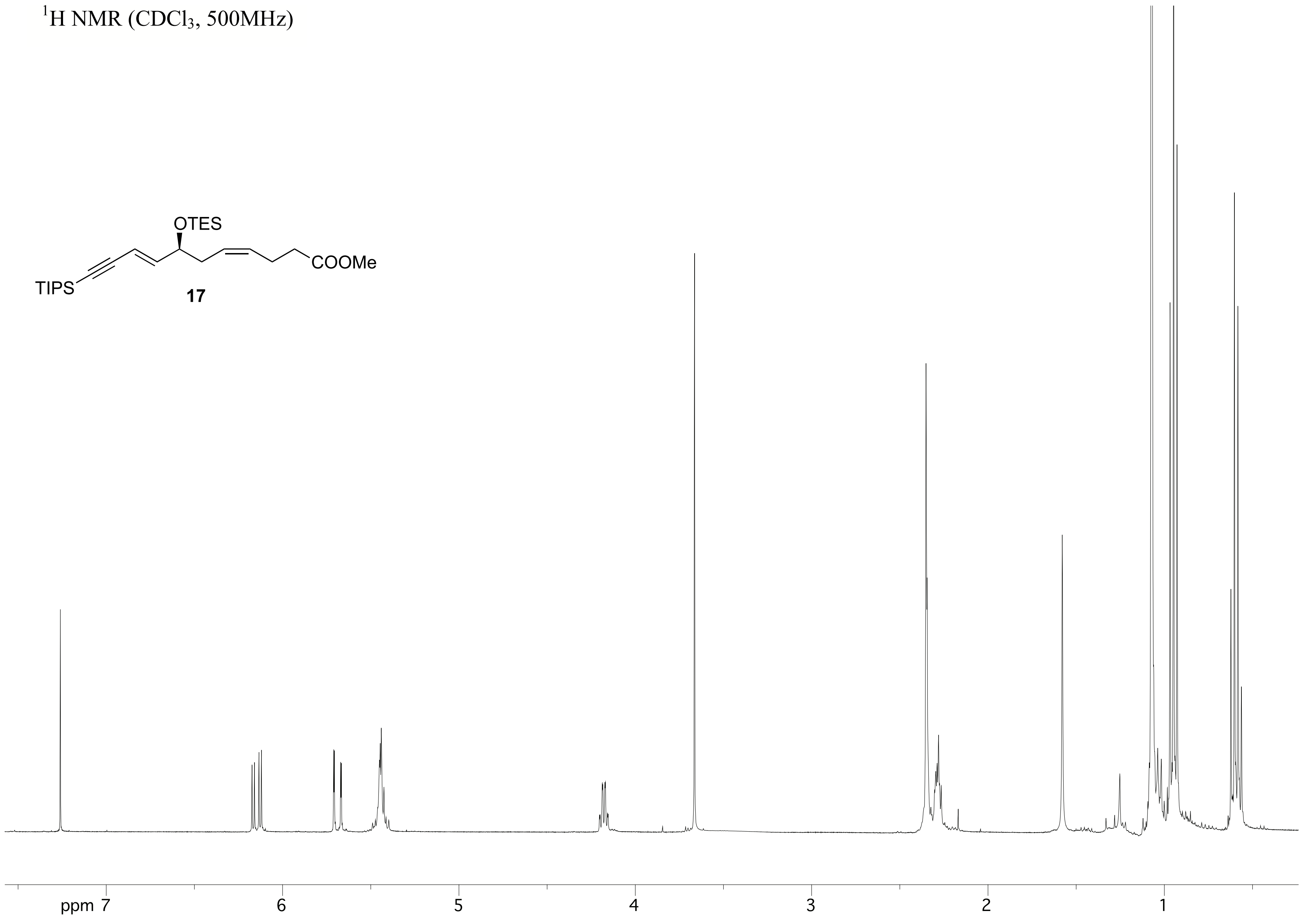

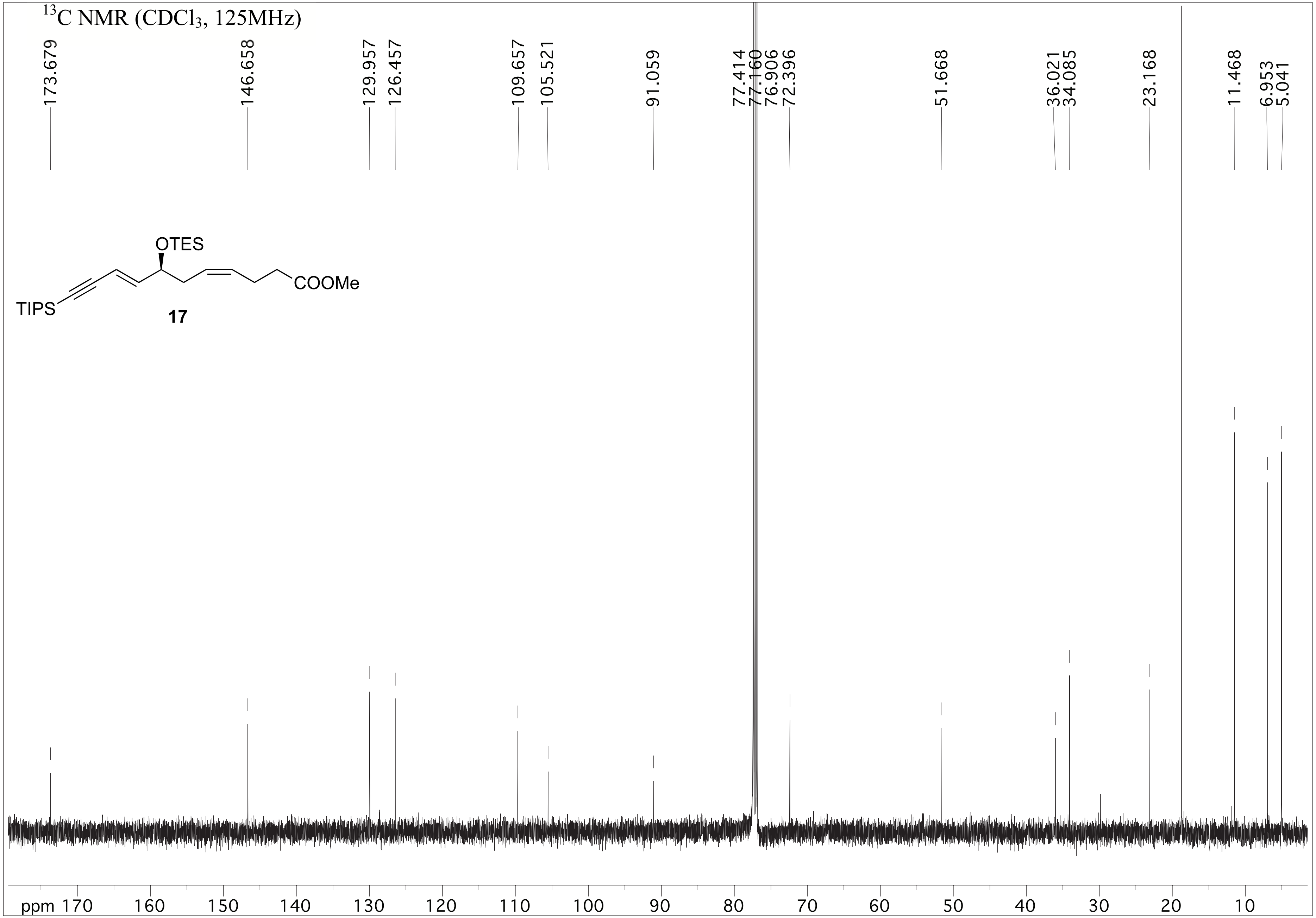
^{13}C NMR ($CDCl_3$, 125MHz)
173.679
146.658
129.957
126.457
109.657
105.521
91.059
77.414
77.160
76.906
72.396
51.668
36.021
34.085
23.168
11.468
6.953
5.041
OTES
TIPS
COOMe
17
ppm 170 160 150 140 130 120 110 100 90 80 70 60 50 40 30 20 10

^{1}H NMR ($CDCl_3$, 500MHz)

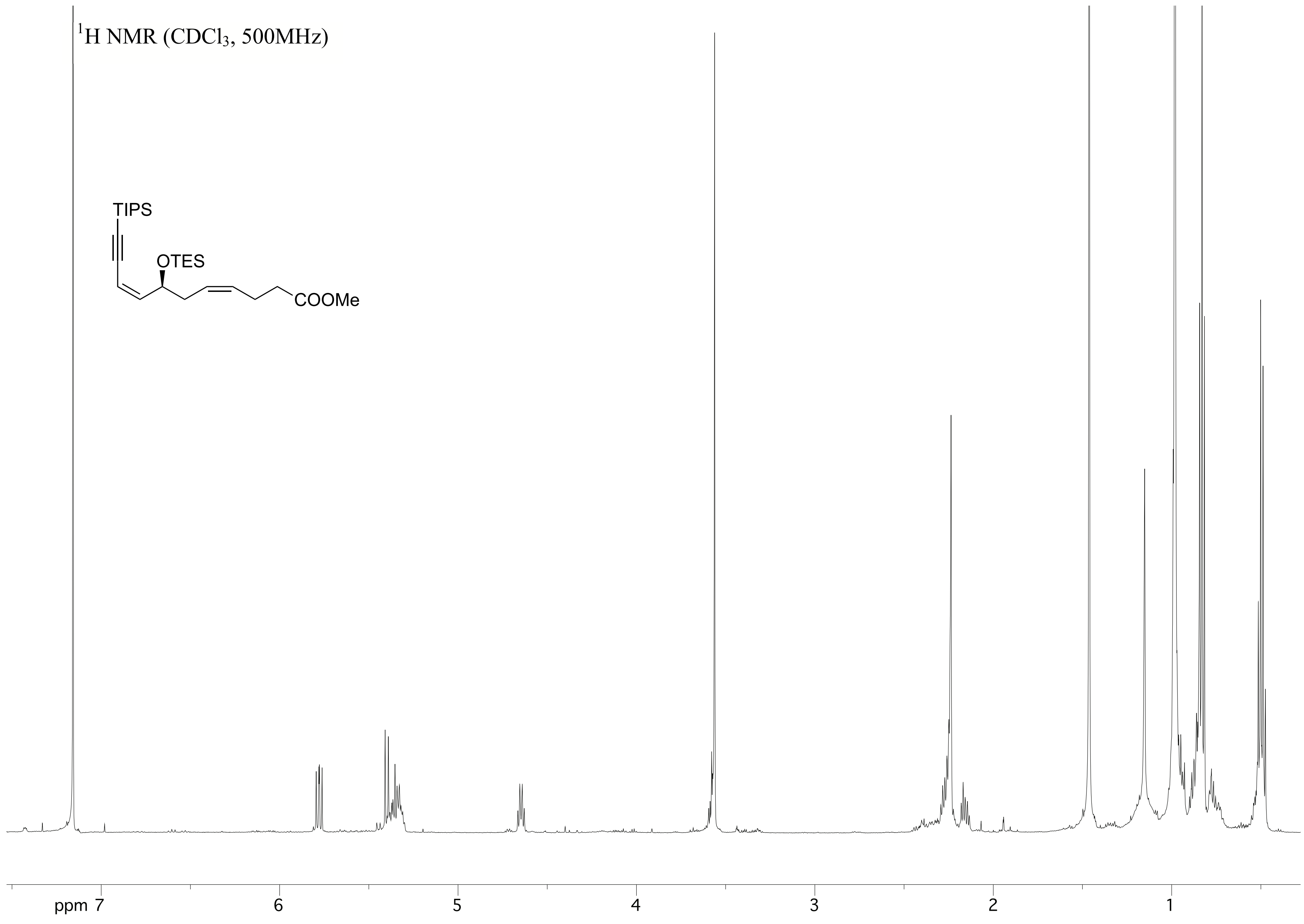

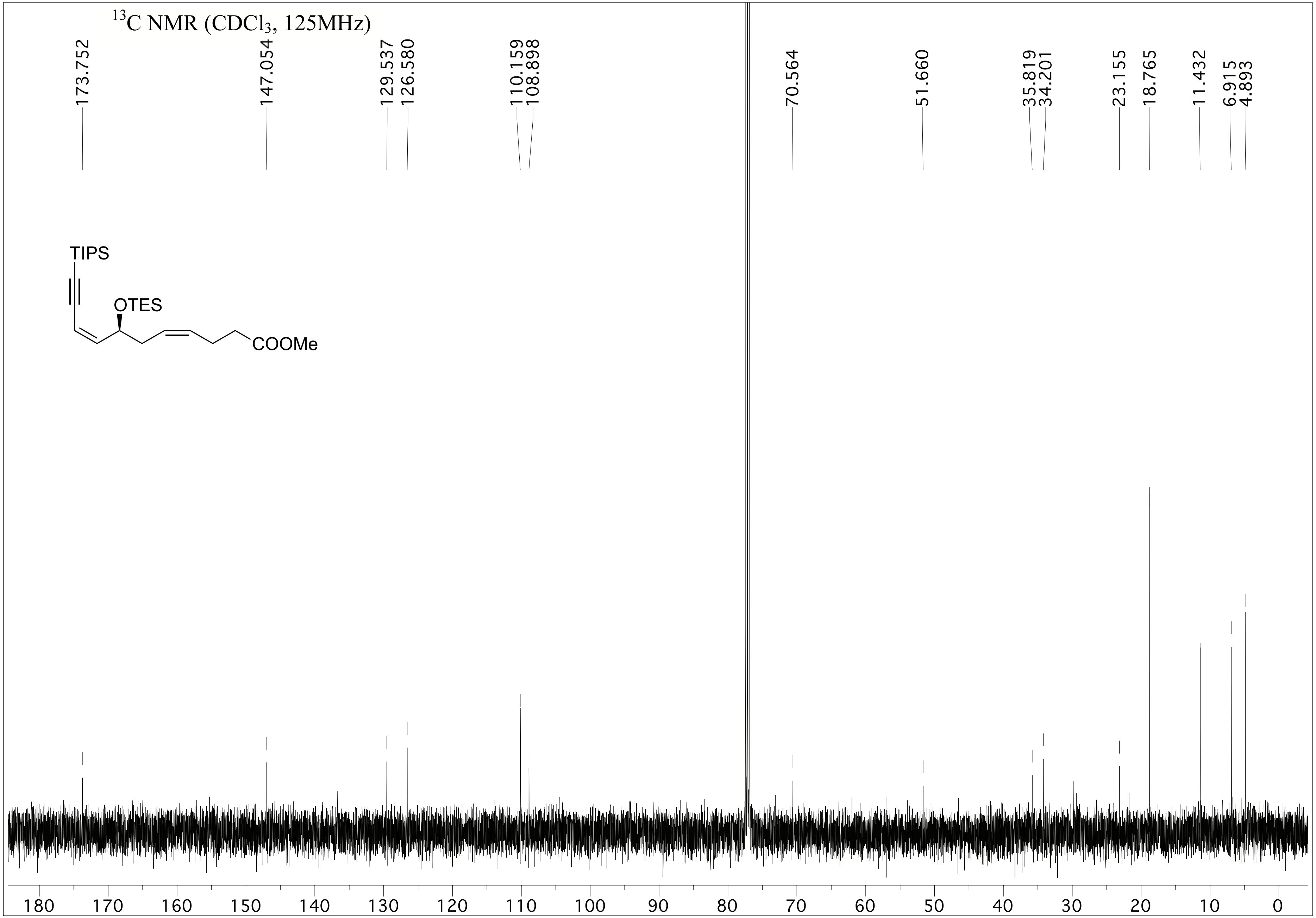

^{13}C NMR (CDCl$_3$, 125MHz)
173.752
147.054
129.537
126.580
110.159
108.898
70.564
51.660
35.819
34.201
23.155
18.765
11.432
6.915
4.893
TIPS
OTES
COOMe
180 170 160 150 140 130 120 110 100 90 80 70 60 50 40 30 20 10 0

^{1}H NMR ($CDCl_3$, 500MHz)

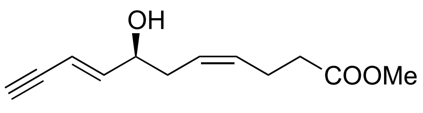

3

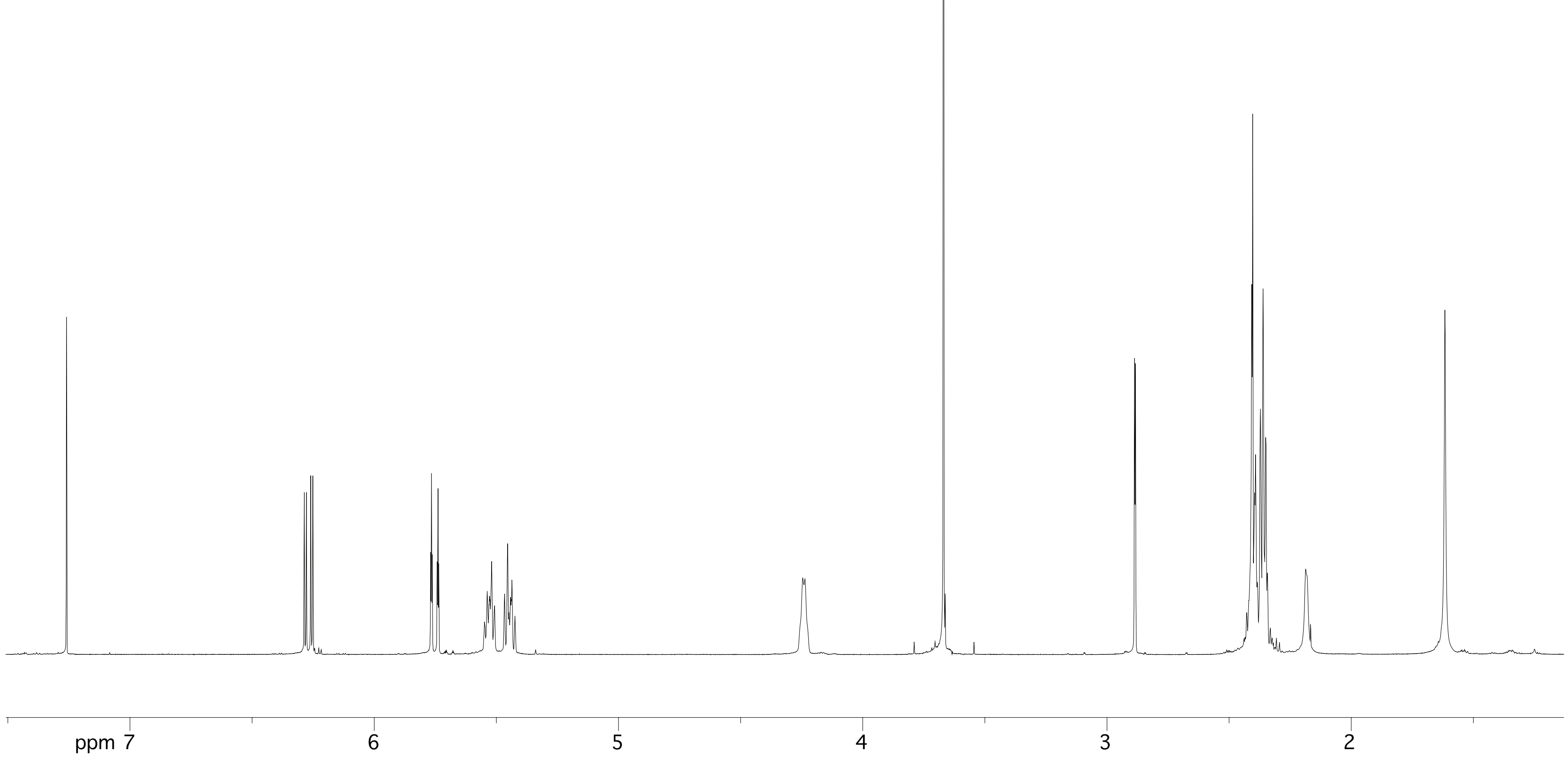

^{13}C NMR ($CDCl_3$, 125MHz)

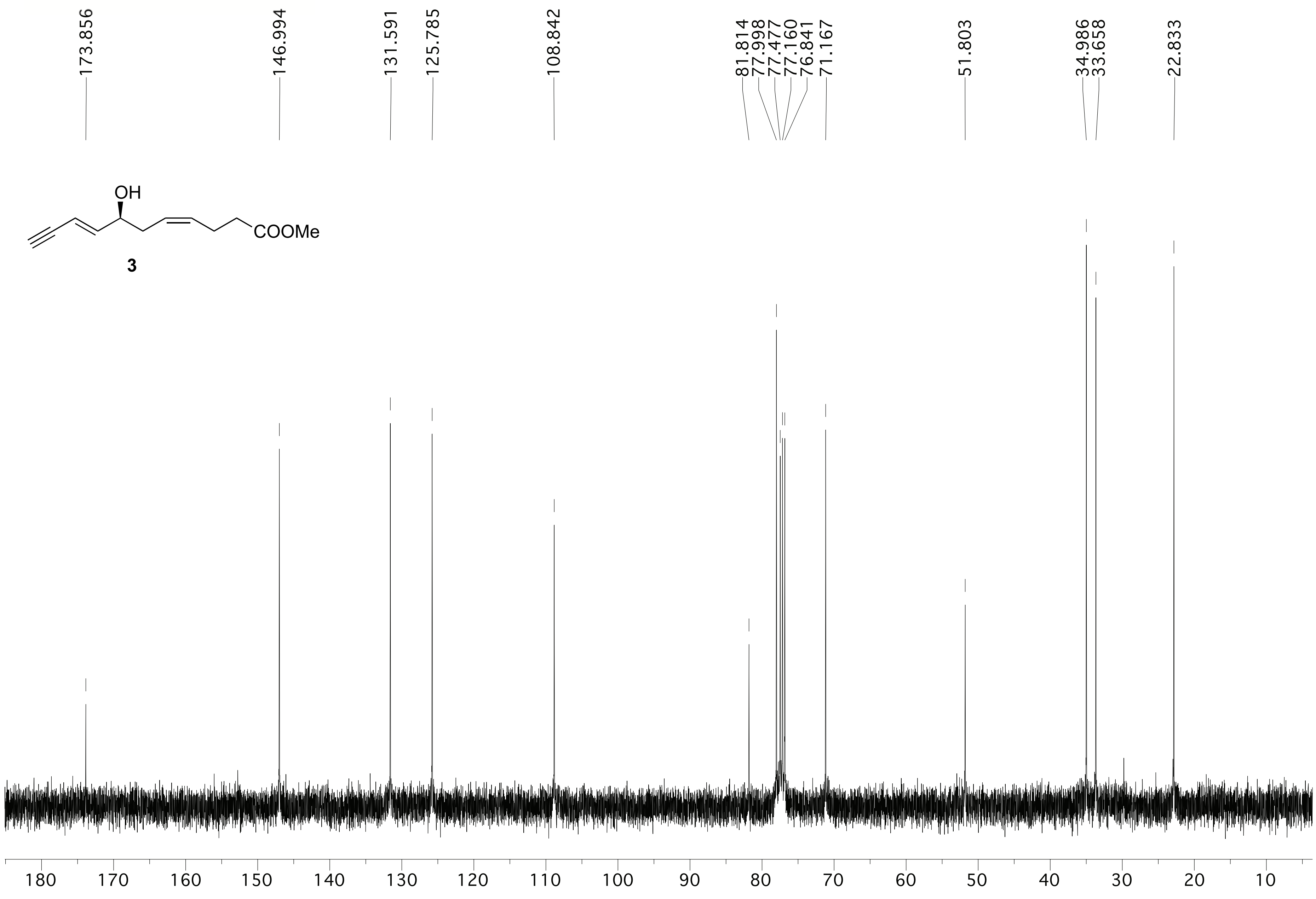

3

^{1}H NMR ($CDCl_3$, 500MHz)

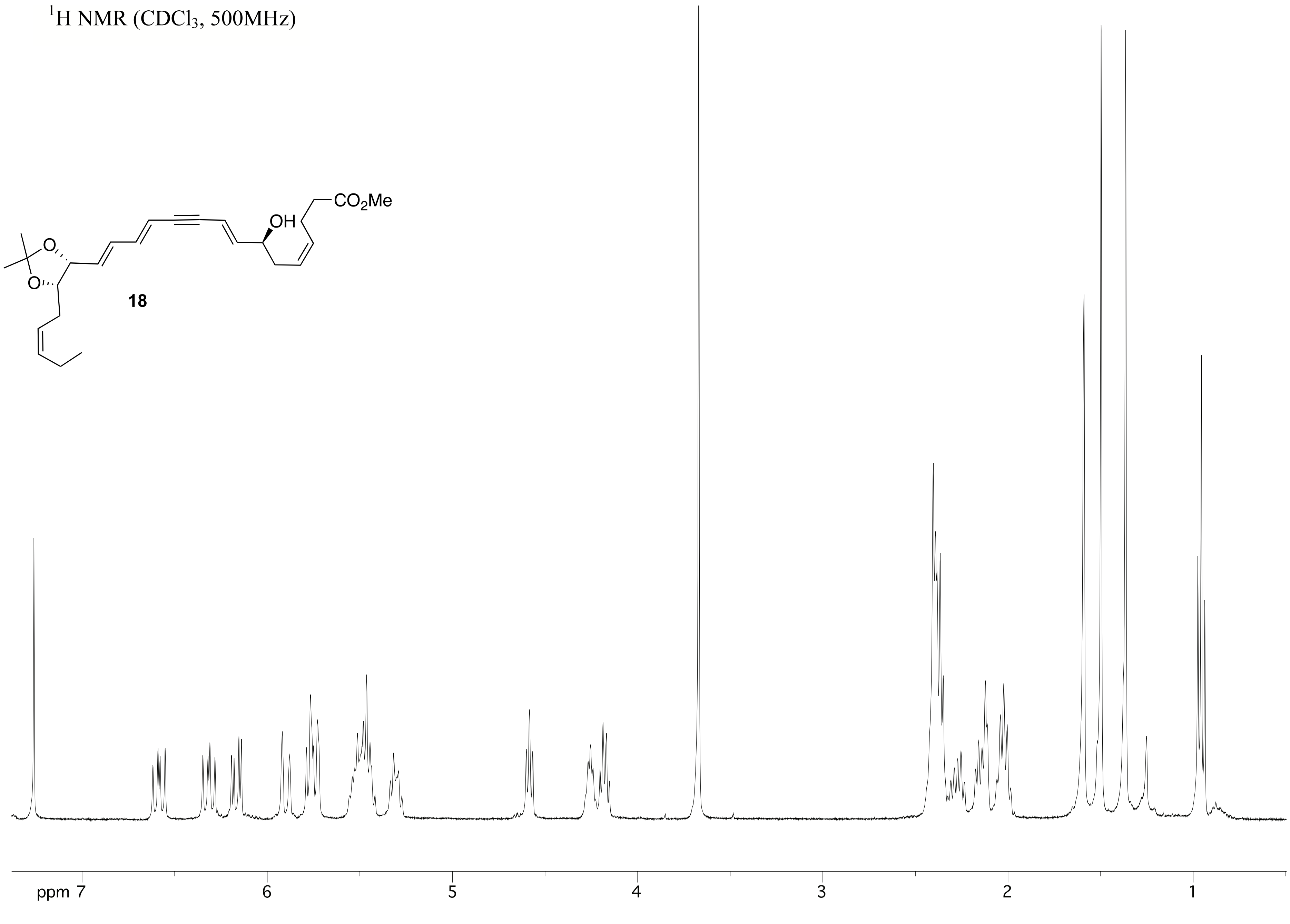

^{13}C NMR ($CDCl_3$, 125MHz)

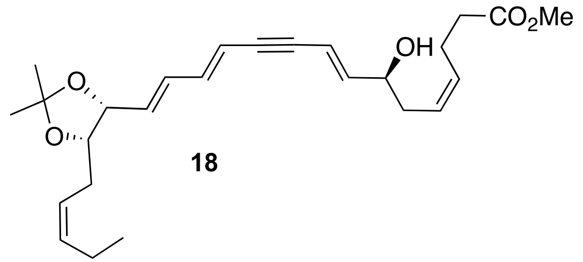

18

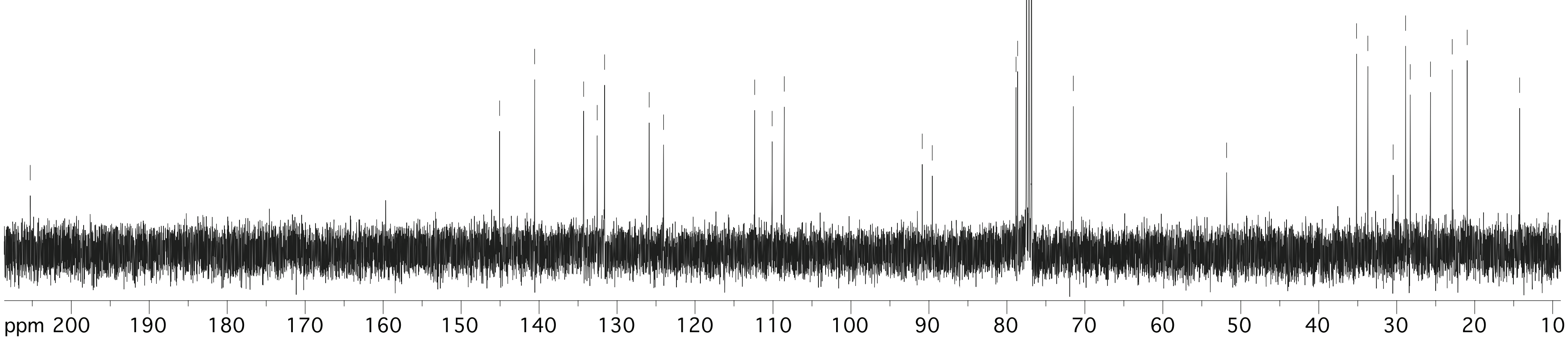

^{1}H NMR ($CDCl_3$, 500MHz)

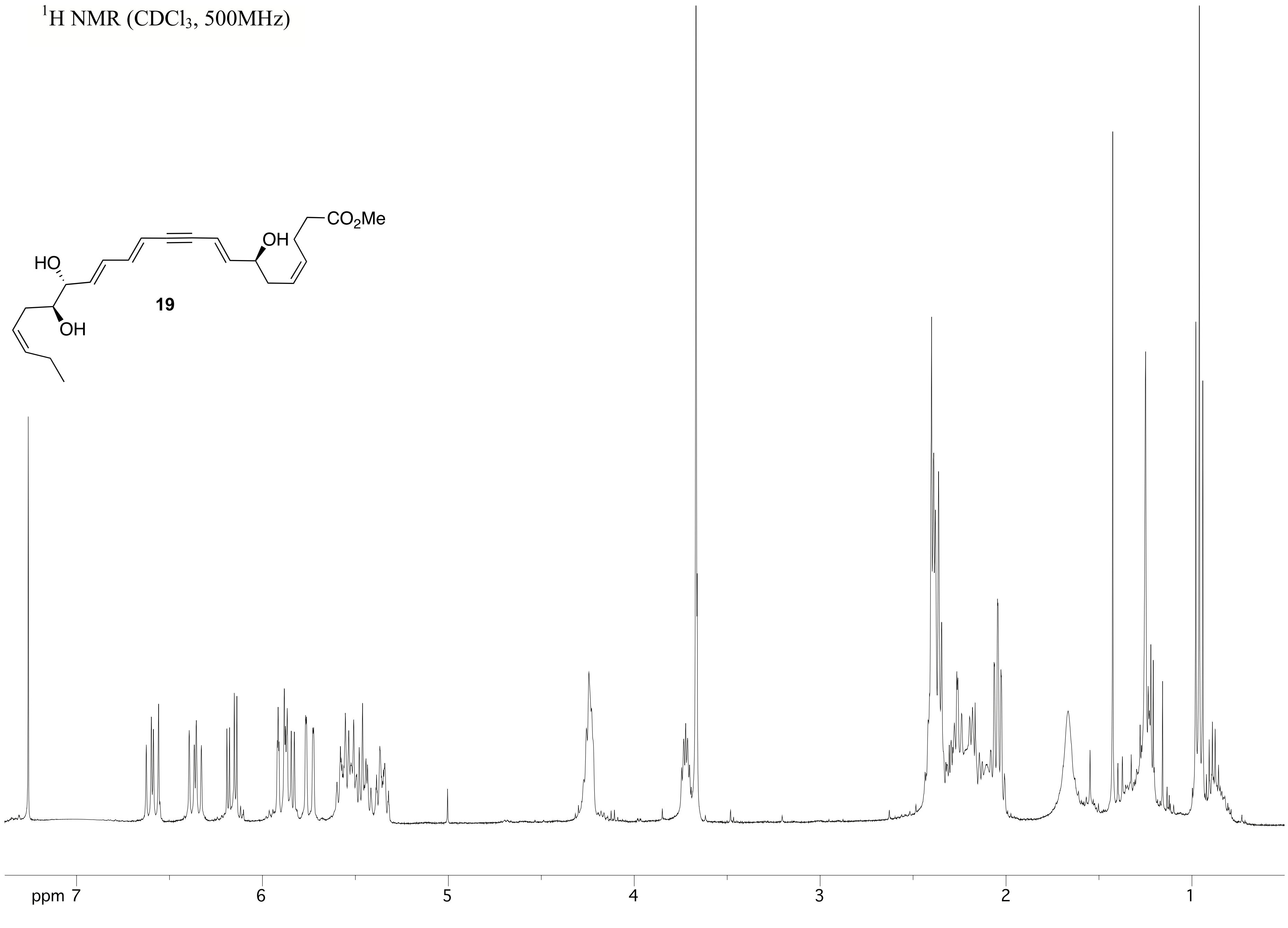

^{13}C NMR ($CDCl_3$, 150MHz)

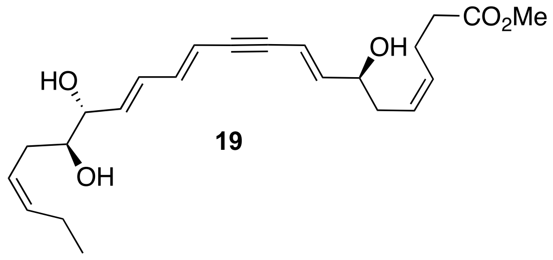

19

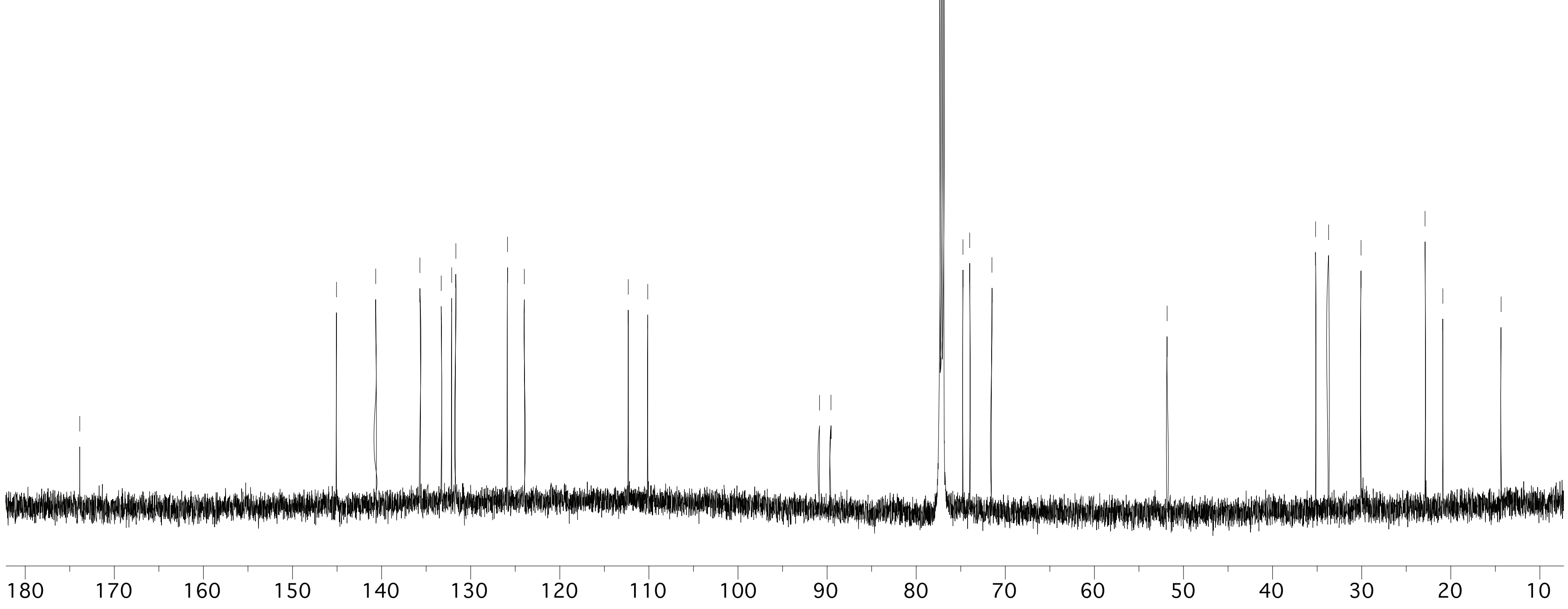

^1H NMR ($CDCl_3$, 400MHz)

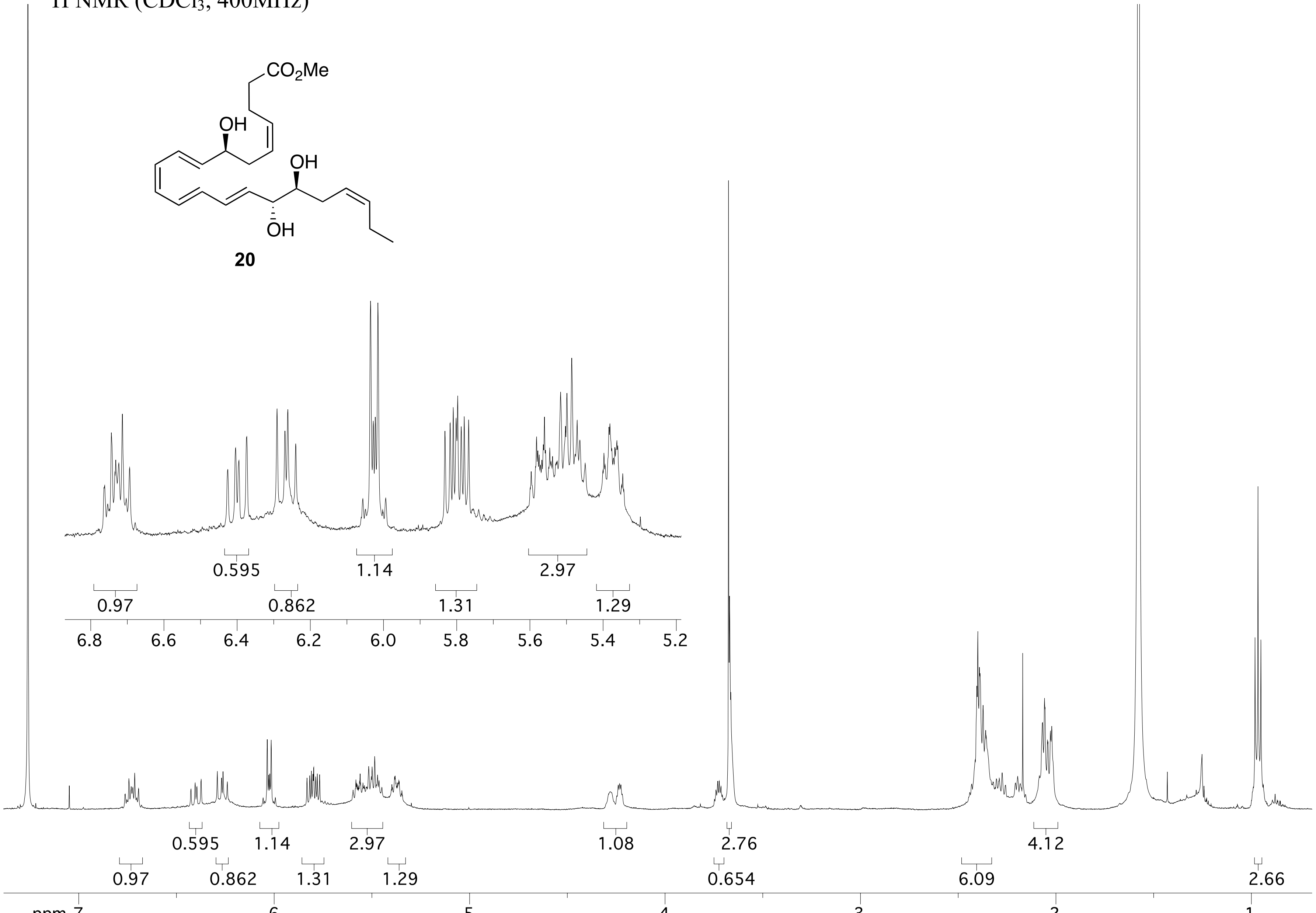

20

^{13}C NMR ($CDCl_3$, 100MHz)

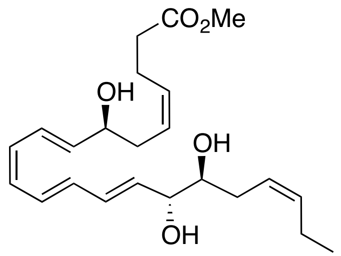

20

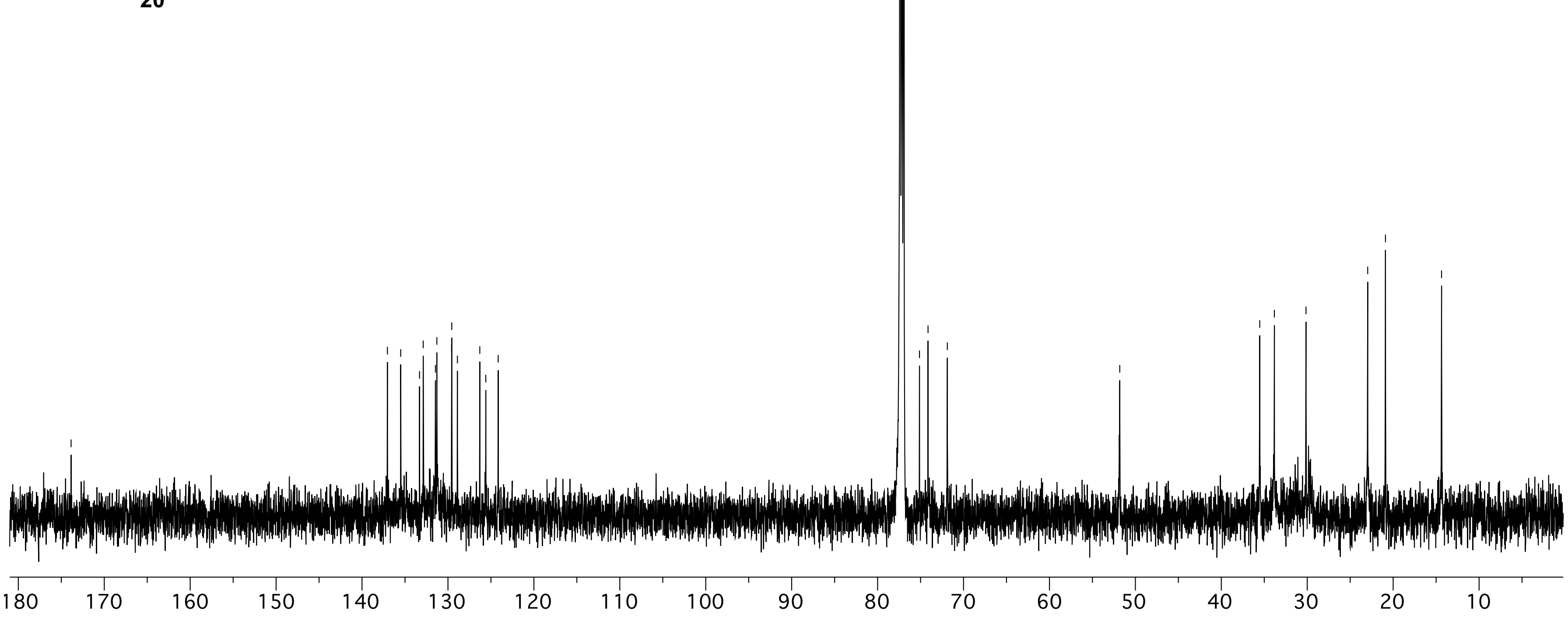

^{1}H NMR (CD_3CN, 500MHz)

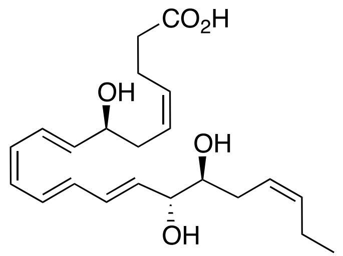

1 Resolvin D2

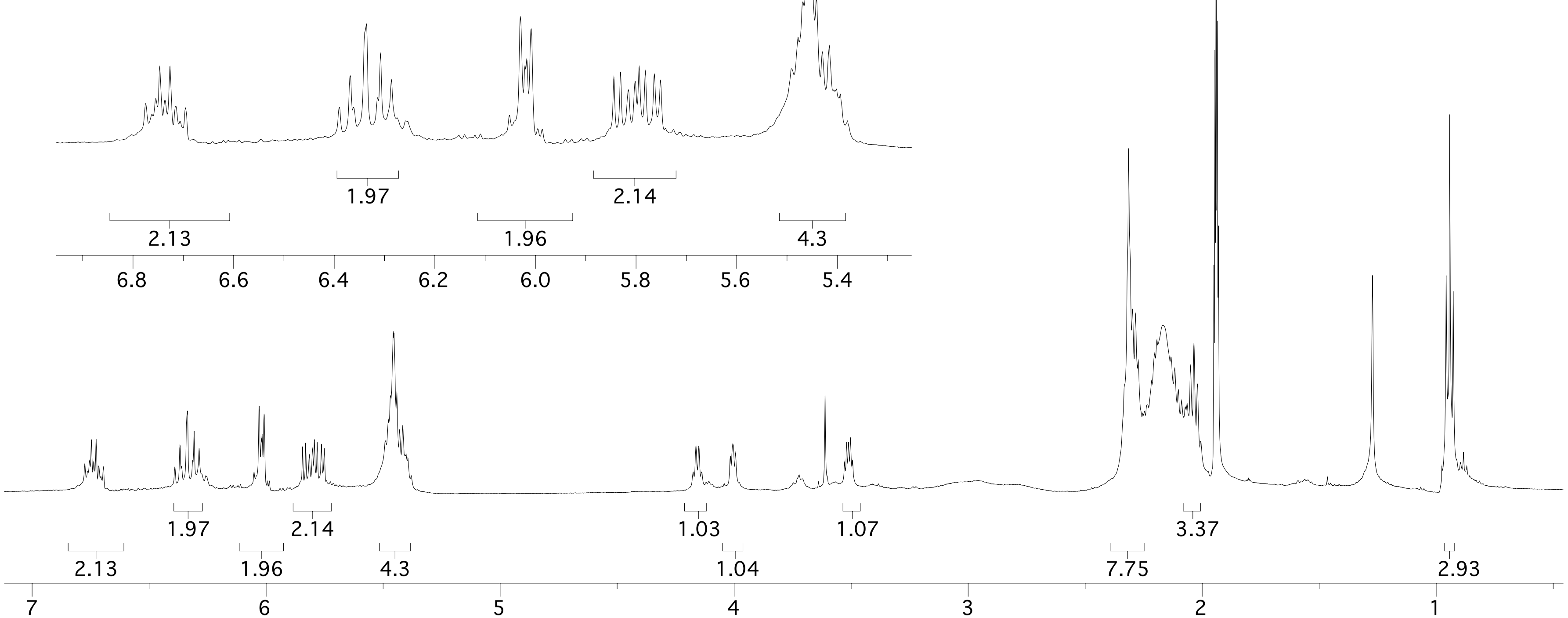

^{13}C NMR (CD_3CN, 125MHz)

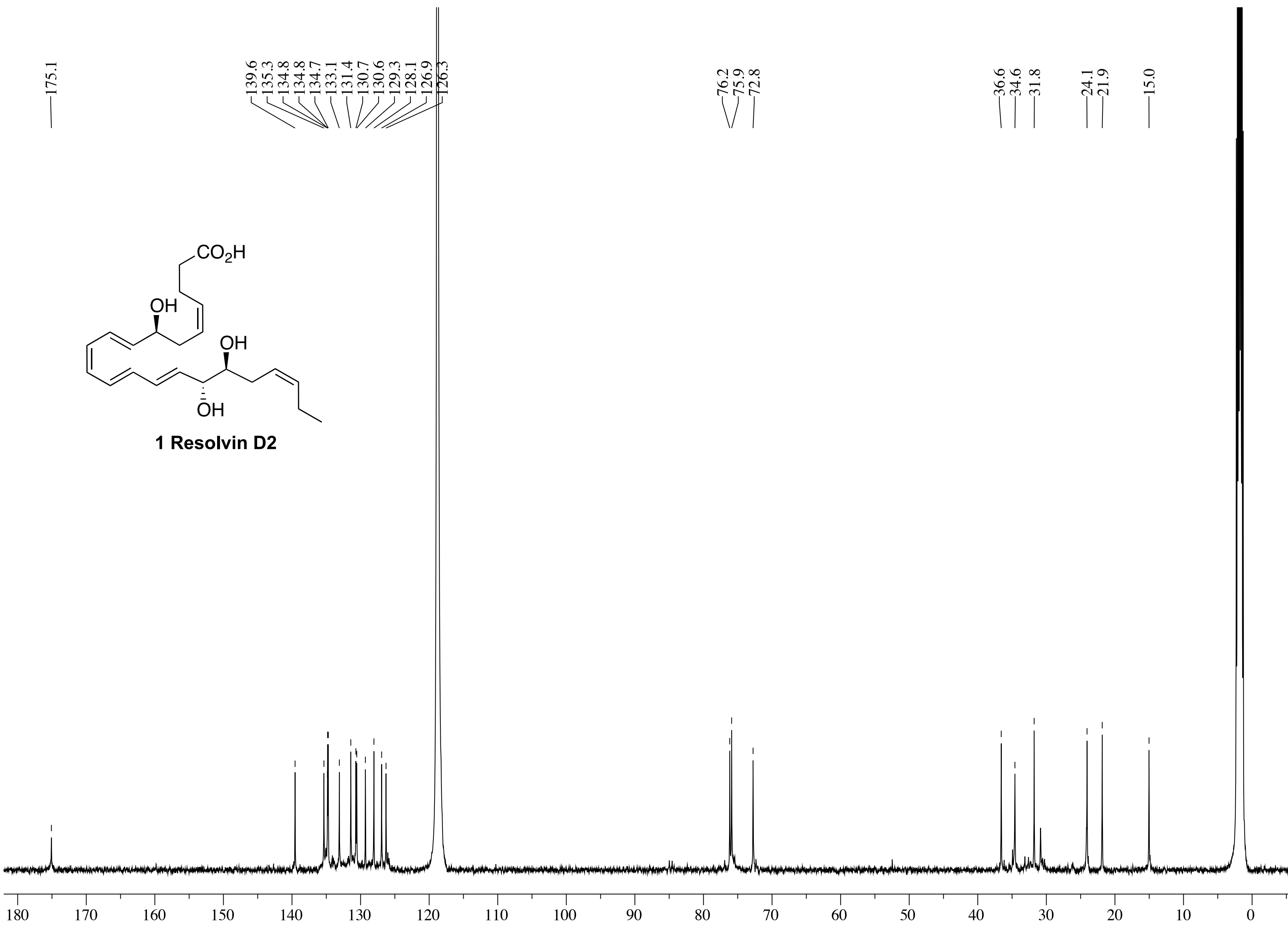

1 Resolvin D2

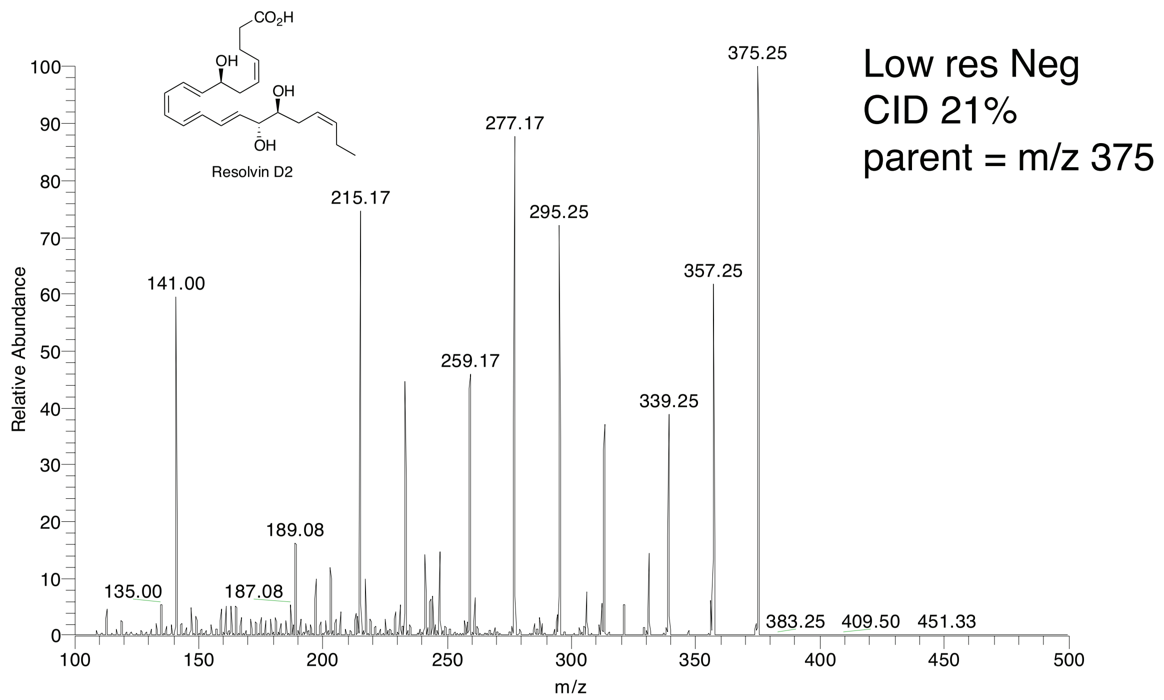
CO2H
OH
OH
OH
Resolvin D2
Relative Abundance
m/z
375.25
357.25
339.25
295.25
277.17
259.17
215.17
189.08
187.08
141.00
135.00
383.25
409.50
451.33
Low res Neg
CID 21%
parent = m/z 375
